# Ape1 guides DNA repair pathway choice that is associated with drug tolerance in glioblastoma

**DOI:** 10.1038/s41598-017-10013-w

**Published:** 2017-08-29

**Authors:** Thomas Ströbel, Sibylle Madlener, Serkan Tuna, Sarah Vose, Tonny Lagerweij, Thomas Wurdinger, Klemens Vierlinger, Adelheid Wöhrer, Brendan D. Price, Bruce Demple, Okay Saydam, Nurten Saydam

**Affiliations:** 1 Institute of Neurology, Medical University of Vienna, A-1090 Vienna, Austria; 2 Molecular Neuro-Oncology Research Unit, Department of Pediatrics & Adolescent Medicine, Medical University of Vienna, A-1090 Vienna, Austria; 3 Vermont Department of Public Health, 108 Cherry St., Burlington, VT 05402 USA; 4 Neuro-Oncology Research Group, Department of Neurosurgery, VU University Medical Center, Amsterdam, 1081 HV Amsterdam, The Netherlands; 5 Department of Neurology, Massachusetts General Hospital, Harvard Medical School, Charlestown, MA 02129 USA; 6 Molecular Diagnostics, AIT - Austrian Institute of Technology, A-1190 Vienna, Austria; 7 Department of Radiation Oncology, Division of Genomic Instability and DNA Repair, Dana-Farber Cancer Institute, Harvard Medical School, Boston, MA 02115 USA; 8 Department of Pharmacological Sciences, Stony Brook University, School of Medicine, Stony Brook, NY 11794-8651 USA

## Abstract

Ape1 is the major apurinic/apyrimidinic (AP) endonuclease activity in mammalian cells, and a key factor in base-excision repair of DNA. High expression or aberrant subcellular distribution of Ape1 has been detected in many cancer types, correlated with drug response, tumor prognosis, or patient survival. Here we present evidence that Ape1 facilitates BRCA1-mediated homologous recombination repair (HR), while counteracting error-prone non-homologous end joining of DNA double-strand breaks. Furthermore, Ape1, coordinated with checkpoint kinase Chk2, regulates drug response of glioblastoma cells. Suppression of Ape1/Chk2 signaling in glioblastoma cells facilitates alternative means of damage site recruitment of HR proteins as part of a genomic defense system. Through targeting “HR-addicted” temozolomide-resistant glioblastoma cells via a chemical inhibitor of Rad51, we demonstrated that targeting HR is a promising strategy for glioblastoma therapy. Our study uncovers a critical role for Ape1 in DNA repair pathway choice, and provides a mechanistic understanding of DNA repair-supported chemoresistance in glioblastoma cells.

## Introduction

Spontaneous hydrolytic decay of DNA or DNA glycosylase-mediated hydrolysis of N-glycosyl bonds generates apurinic/apyrimidinic (AP) sites at an estimated frequency of >10,000 per day in each human cell^1^. When AP sites are bypassed by replication or translesion DNA polymerases, mutagenesis ensues^2^. Thus, the proper repair of AP sites is crucial for maintaining genome stability. The predominant human AP endonuclease 1, Ape1 (also known as Ref-1, and encoded by the *APEX1* gene) produces a nick at the AP site and allow for further processing of DNA^3–11^. In addition, Ape1 exhibits 3′-5′ exonuclease, 3′-phosphatase, and 3′-phosphodiesterase activities^8–11^. These additional activities of Ape1 remove 3′-end blocking groups produced by radiation, ROS, or other DNA glycosylases^12^. The multifunctional protein Ape1 also functions as a redox^13^ and a transactivator protein^14, 15^, in addition to its role in RNA quality^16^. A vital role for Ape1 in mammalian cell viability has been shown^17–21^, yet, the precise mechanism(s) in this requirement is not well understood. In this study, we discovered an additional, unpredicted role for Ape1 in DNA repair pathway choice. We found that Ape1 facilitates homologous recombination (HR), and counteracts error-prone non-homologous end joining (NHEJ) of DNA ends. Furthermore, in collaboration with a DNA damage checkpoint kinase Chk2, Ape1 regulates the chemotherapy response of glioblastoma cells. Thus, our study uncovers a unique Ape1-mediated genome maintenance mechanism, and highlights its importance in cancer chemoresistance.

## Results

### Ape1 and Chk2 cooperate to ensure genomic stability during replication stress

**Figure 1:**
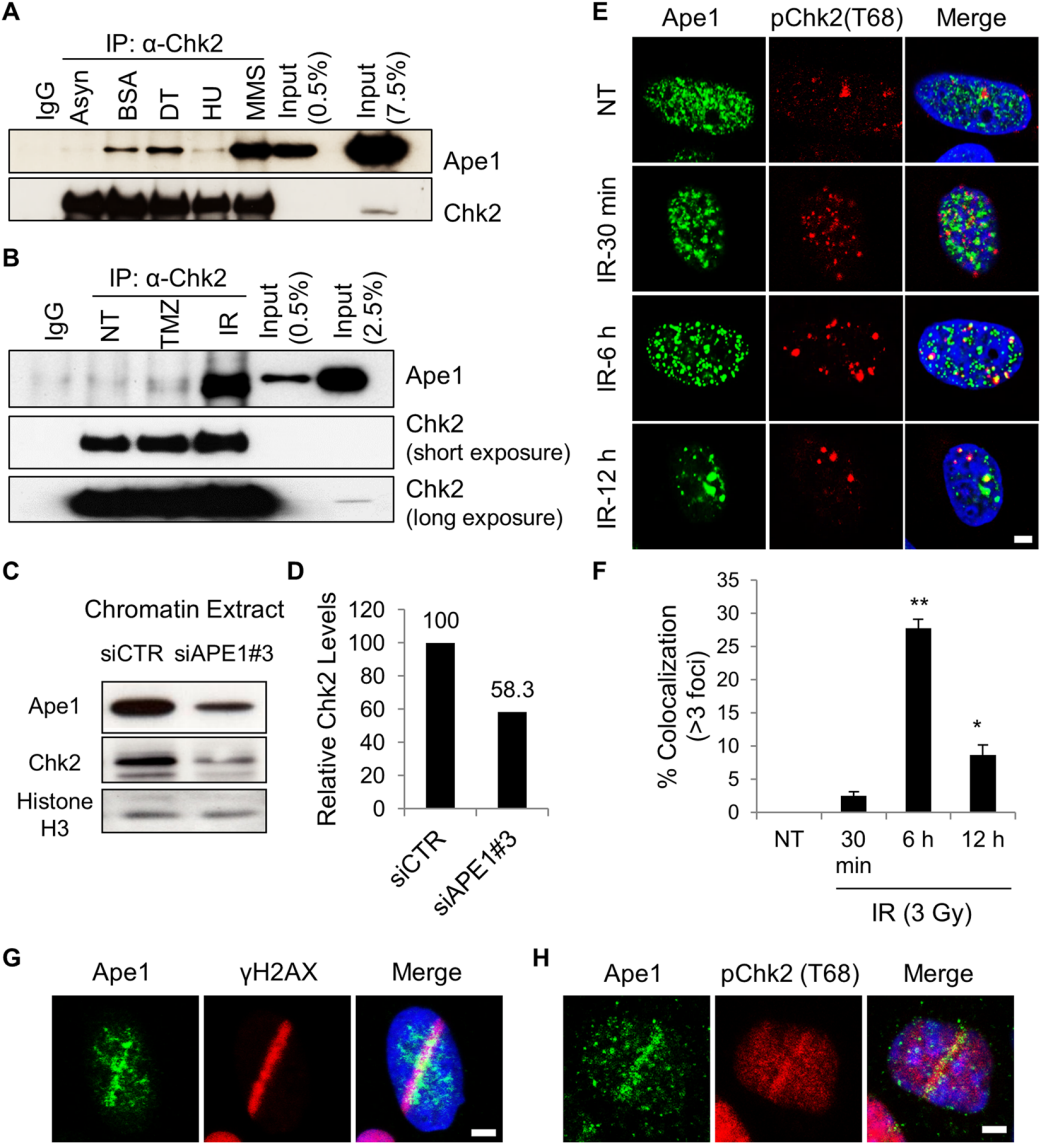
Ape1 is recruited to sites of double-strand breaks of DNA, and cooperates with Chk2. **(A)** HEK293 cells were treated with BSA (0.5%; 24 h), double thymidine (DT, 2 mM), hydroxyurea (HU, 2 mM; 16 h), or methyl methane sulphonate (MMS, 250 µM; 4 h). MMS treated cells were washed and incubated for another 16 h. Asynchronized cells were left untreated. Cells were then harvested, and subjected to IP. **(B)** HEK293 cells were either left non-treated (NT), exposed to temozolomide (TMZ, 250 µM, 4 h) or ionizing radiation (IR, 3 Gy). TMZ-treated cells were washed and incubated for another 4 h prior to the harvest. IR-treated cells were harvested 6 h after the IR exposure. Cells were then subjected to IP, followed by immunoblotting for Ape1 and Chk2 proteins. **(C)** Chromatin fractions were isolated after 72 h of siRNA transfections, and subjected to Western blot. **(D)** Quantification of relative Chk2 signal at chromatin is shown. **(E)** U2OS cells were exposed to IR (3 Gy), fixed and stained with the indicated antibodies. Representative images are shown. Scale bar, 5 µm. **(F)** At least 100 cells per experiment were counted, and cells with at least three colocalizing foci were quantified. Standard deviations were calculated from three independent experiments. **p < 0.001 (IR, 30 min vs. IR, 6 h), *p < 0.02 (IR, 30 min vs. IR, 12 h); Student’s *t* test **(G)** U2OS cells were micro-irradiated, fixed after 2 min of laser incision, and stained with Ape1 and γH2AX antibodies. A representative image is presented from at least three independent micro-irradiation experiments. Scale bar, 5 µm. **(H)** U2OS cells were micro-irradiated, fixed, and stained with Ape1 and phospho-Chk2 (Thr 68). A representative image is presented from at least three independent micro-irradiation experiments. Scale bar, 5 µm.

We have previously reported that Ape1-depleted cells exit mitosis with chromosomal defects, and display mitotic failure, pointing to the defects in DNA damage checkpoint control^22^. The response of Ape1-depleted cells to DNA damaging agents further confirmed that Ape1 is required for efficient checkpoint control in response to genomic stress. To further address molecular mechanisms of defective checkpoint control in Ape1-depleted cells, we examined the major checkpoint signaling pathways, ATR/Chk1 and ATM/Chk2^23^ for their possible impact on the Ape1-associated cell responses, and found that Ape1 and Chk2 associate in HEK293 cells upon replication stress (Fig. 1A,B; Fig. S1A,B). Notably, methyl methane sulfonate (MMS), which generates methylated bases and AP sites in cellular DNA^24^ stimulated *in vivo* association of Ape1 and Chk2 in the cell, while temozolomide (TMZ), another DNA alkylating agent that is used in clinic for cancer chemotherapy, induced relatively weaker complex formation. In addition, exposure to ionizing radiation stimulated association of Ape1 and Chk2 in HEK293 cells. The treatments such as thymidine, BSA and HU causing an arrest at the G1/S transition in the cell cycle also slightly induced the complex formation of Ape1 and Chk2, which may reflect replication stress-related DNA damages caused by these agents. We also found that chromatin-bound form of Chk2 protein was reduced in Ape1-siRNA treated cells (Fig. 1C,D; Fig. S2A–C), which was consistent with reduced levels of Chk2 in total cell extracts of Ape1-depleted U2OS cells (Fig. S3A–C). On the other hand, the depletion of Ape1 in our experimental setting did not cause Chk1 phosphorylation at serine-345, a target of ATR^25^ (Fig. S3D). These results suggest that Ape1 and Chk2 form a functional protein complex in the cell. Our cycloheximide chase assay showed that Ape1 is stabilized in response to Chk2 depletion (Fig. S4A), and Ape1 is required for the stabilization of Chk2 in response to temozolomide (Fig. S4B), suggesting that the stability of Ape1 and Chk2 proteins are dynamically regulated by each other.

### Ape1 plays a role in DSB repair of DNA

In response to ionizing radiation (IR), Chk2 is rapidly phosphorylated at threonine 68 (Thr 68) by ATM, and this activated form of Chk2 localizes at sites of DNA strand breaks^26^. To address whether Ape1 depletion has an effect on ATM/Chk2 signaling, we exposed Ape1-depleted U2OS to IR, and tracked the phosphorylation of Chk2 at threonine 68 position (Thr 68) for a period of 12 h. IR caused a rapid pChk2 (Thr 68) focus formation in U2OS cells, while Ape1 was juxtapositioned with the foci of pChk2 at 30 min after IR (Fig. 1E). The IR-induced colocalization of pChk2 (Thr 68) and Ape1 was apparent at 6 h of the exposure, and the signal was gradually decreased at the later time points (Fig. 1E,F). These results suggest that Ape1 functionally associates with Chk2 in response to double-strand breaks (DSBs) of DNA pointing to a previously unknown role for Ape1 at the repair of DSBs.

To further validate the recruitment of Ape1 to the sites of DSBs, we micro-irradiated U2OS cells, and tracked endogenous γH2AX and Ape1 within several minutes of micro-irradiation. The data showed that Ape1 coincides with γH2AX, a marker of DNA DSB damage at the micro-irradiated sites of DNA (Fig. 1G). In addition, Ape1 appeared to overlap with pChk2 (Thr 68) signal at the site of laser incision (Fig. 1H). Thus, these data support the idea that Ape1 is recruited to DSB sites of DNA, and *in vivo* association of Ape1 and Chk2 indicates a functional role during DSB repair of DNA.

To investigate the functional implications of the Ape1/Chk2 cross-talk for human cancer etiology, we performed a search using The Cancer Genome Atlas (TCGA)^27, 28^, which revealed that the patients with glioblastoma who express higher levels of *APEX1* have a better survival rate (R2: Microarray Analysis and Visualization Platform; Kaplan-Meier Scanner) (Fig. S3E).

In addition, the co-expression of *APEX1* and *CHEK2* genes was positively correlated in the TCGA glioblastoma data set (r = 0.198, p = 3.6.10–6, n = 540; Tumor Glioblastoma-TCGA-540-MAS5.0-u133a dataset; R2: Microarray Analysis and Visualization Platform). These preliminary data obtained through the analyses of TCGA prompted us to choose glioblastoma tumor model for further mechanistic studies.

### Ape1 is rapidly recruited to DSB sites of DNA in glioblastoma cells, and that function of Ape1 does not require an intact AP endonuclease domain

**Figure 2:**
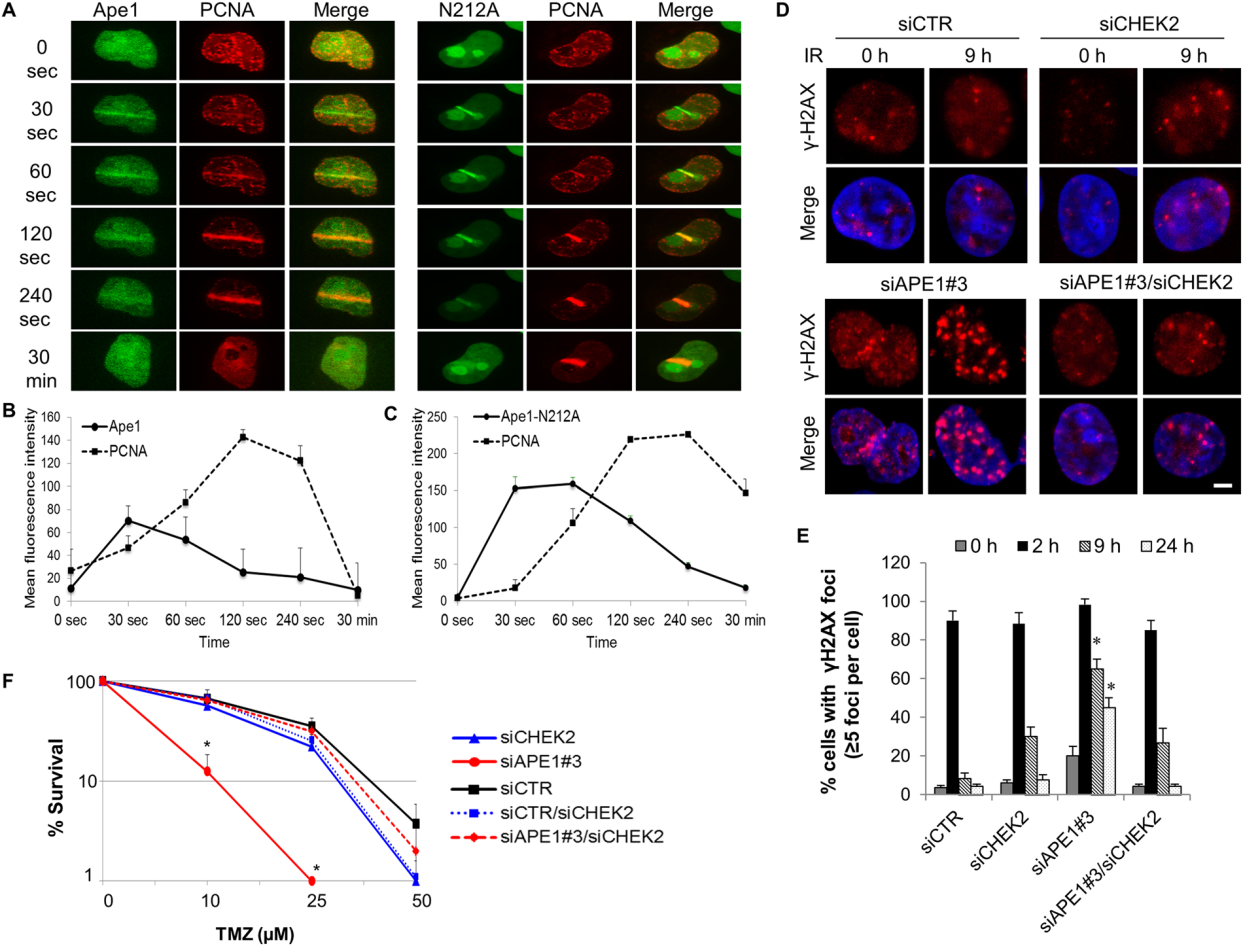
Ape1 depletion results in DNA damage accumulation, and that is counteracted by concomitant depletion of Chk2 in glioblastoma cells. **(A)** U251-MG cells were exposed to laser micro-irradiation, and green (Ape1) and red (PCNA) fluorescence were live-imaged for 30 min. At least three independent irradiations were performed for each cell type. Representative images are shown. **(B)** Time-dependent damage site recruitments of Ape1, **(C)** Ape1-N212 A, and PCNA were determined as described in Methods. **(D)** U251-MG cells were exposed to IR (3 Gy) at 48 h of siRNA transfection. Cells were fixed at the indicated time points, and immunostained for γH2AX. Representative images are shown. Scale bar, 5 µm. **(E)** At least 100 cells per sample were counted from each experiment, and cells showing more than five γH2AX foci were quantified. Error bars indicate standard deviations obtained from three independent experiments. *p < 0.01 (siAPE1#3 vs. siAPE1#3/siCHEK2); Student’s *t* test. **(F)** U251-MG cells were transfected with the indicated siRNAs, and colony formation assay was performed after TMZ exposure. Data were obtained from three independent experiments. Error bars indicate standard deviation. *p < 0.05 (siAPE1#3 vs. siAPE1#3/siCHEK2); Student’s *t* test.

To validate the recruitment of Ape1 to the sites of DSBs in glioblastoma cells, U251-MG cells expressing a GFP-tagged wild-type Ape1 along with a RFP-tagged PCNA^29^ were laser micro-irradiated, and the intensity of fluorescence was recorded in living cells for 30 min. The recruitment of wild-type Ape1 to the damage site occurred within seconds of laser induction of DSBs, and reached to the highest intensity at 30 s (Fig. 2A,B). The dissociation of Ape1 from the damaged site was noticeable after 60 s, and completed at 30 min. The dynamics of PCNA recruitment were slower, reaching to the peak intensity at 120 s, and mostly dissociating by 30 min after the laser micro-irradiation (Fig. 2A,B). To investigate which domain(s) of Ape1 is required for the recruitment to DSB sites, we tracked the Ape1 variants defective for base-excision repair (Ape1-N212A), redox regulatory (Ape1-C65S), or transcriptional activator function (Ape1-K6A/K7A) following the laser micro-irradiation. The data showed that the mutations targeting the functional domains of Ape1 did not prevent the recruitment of Ape1 to the laser micro-irradiated sites of DNA (Fig. 2A,C; Fig. S5A,B). These results suggest that the recruitment of Ape1 to DSB sites is a rapid process, and is independent of AP endonuclease-, redox-, or transcriptional regulation-related activities of Ape1.

### Depletion of Ape1 causes IR-induced DNA damage accumulation, which is prevented by depletion of Chk2

To address the role of Ape1 in DSB repair, we visualized the dynamics of DSB repair in Ape1- and/or Chk2-depleted U251-MG cells by tracking γH2AX, a DNA damage marker^30–32^. Our results showed that Ape1-depleted cells retained substantial DNA damage at 9 h and 24 h after IR (Fig. 2D,E). In contrast, the cells depleted for both Ape1 and Chk2 were capable of repairing IR-induced damages at 9 h of the IR exposure (Fig. 2D,E). These results suggest that Ape1 is required for the repair of IR-induced damages of DNA, and the accumulation of DNA damage caused by Ape1 deficiency is counteracted by Chk2 deficiency.

### Ape1 deficiency increases TMZ responsiveness in glioblastoma cells, which is reversed by concurrent depletion of Chk2

Depletion of Ape1 causes cell proliferation defects *in vitro* (Fig. S6A). To explore the role of Chk2 in Ape1-mediated cellular responses in glioblastoma tumor model, we exposed U251-MG cells to temozolomide, a DNA alkylating agent that is currently used as an effective adjuvant therapy in glioblastoma patients^33^, and observed that clonogenic survival of Ape1-depleted cells was markedly reduced in response to TMZ (Fig. 2F). This effect was expected, because TMZ alkylates DNA bases, and generates substrates for Ape1 activity during BER^34^. Thus, a deficiency in Ape1 is expected to hinder the repair of base damages, and potentiate the cytotoxic effects of TMZ. However, the depletion of both Chk2 and Ape1 together counteracted the Ape1deficiency-associated cytotoxicity of TMZ, and sustained the cell survival (Fig. 2F). By employing an orthotopic glioblastoma model in mice^35^, we verified that depletion of Ape1 in U251-FM cells caused defective tumor growth, yet, the simultaneous depletion of Ape1 and Chk2 restored the tumor growth in mice (Fig. S6B–D). Supporting these findings, we detected that Ape1/Chk2- depleted U2OS cells were able to incorporate BrdU into their DNA (Fig. S6E), confirming that DNA synthesis resumes in the cells deficient for both Ape1 and Chk2. Thus, these results suggest that Ape1 and Chk2 play coordinated roles in cellular proliferation. Although Ape1 may be a likely substrate of Chk2 kinase, further studies are required for identification of possible Chk2-mediated phosphorylation sites of Ape1, as well as for assessing its functional significance.

### Ape1/Chk2 signaling guides cellular decisions to switch between DNA repair pathways

**Figure 3:**
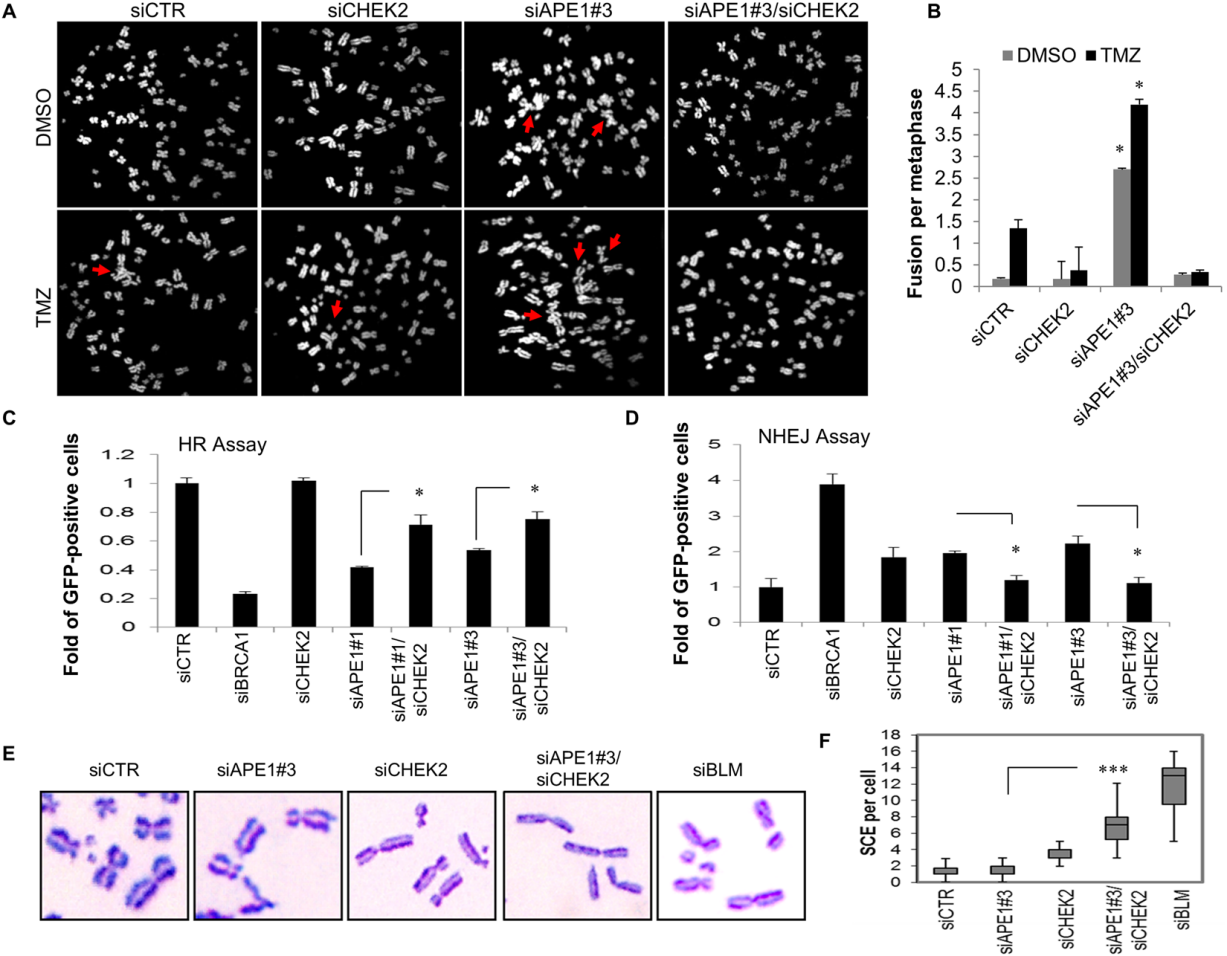
Dual suppression of Ape1 and Chk2 facilitates HR. (**A)** U251-MG cells were transfected with the indicated siRNAs, and treated with TMZ (1000 µM, 1 h) at 48 h of transfection. Metaphase chromosomes were prepared 24 h after exposure to TMZ, and labelled with DAPI. Representative metaphases are shown. Red arrows indicate chromosome fusions. **(B)** At least 50 metaphases per sample were counted, and standard deviations were calculated from three independent experiments. *p < 0.001 (siAPE1#3 vs. siAPE1#3/siCHEK2); Student’s *t* test. **(C)** U2OS-DR cells were transfected with the indicated siRNAs and a plasmid expressing I-Sce I. Percentage of GFP-positive cells was quantified by flow cytometry. Percentage of values scored in the control cells (siCTR) was normalized to 1. Data were obtained from three independent experiments. *p < 0.05; Student’s *t* test. **(D)** H1299dA3-1 cells were transfected with the indicated siRNAs and an I-Sce I vector. Percentage of GFP-positive cells was calculated as described in (**C)**. Data from three independent experiments are shown. *p < 0.05; Student’s *t* test. **(E)** U2OS cells were transfected with the indicated siRNAs, and incubated with BrdU (10 µM) for 24 h. After the treatment with MMS (350 µM) for 3 h, metaphase chromosomes were stained with Giemsa. Representative images are shown. **(F)** At least thirty metaphase cells per sample were analyzed, and medians of the number of chromosomes with SCE were presented in a box plot chart. ***p < 0.0001; (siAPE1#3 vs. siAPE1#3/siCHEK2); Student’s *t* test.

Our study further focused on understanding how the crosstalk between Ape1 and Chk2 impacts on the maintenance of genome stability. Previously, we reported that Ape1 deficiency is associated with telomere defects and chromosome fusions^22^. To examine whether the chromosome fusions caused by Ape1 deficiency can be rescued by Chk2 depletion in the glioblastoma cells, we analyzed the metaphase chromosomes of the Ape1- and/or Chk2-depleted U251-MG cells, and observed that depletion of Ape1 causes massive chromosome fusions in U251-MG cells, and that is further augmented by TMZ (Fig. 3A,B). Consistently, depletion of Chk2, along with Ape1, prevented the formation of chromosome fusions regardless of an exposure to TMZ (Fig. 3A,B). These data support the idea that cells with a suppressed Ape1/Chk2 signaling switch to an alternative DNA repair pathway that counteracts formation of damage-induced chromosome fusions, pointing to a functional connection between Ape1 and Chk2 in glioblastoma drug response.

### Ape1 facilitates homologous recombination and counteracts non-homologous end joining of DNA ends

To identify prevailing DNA repair pathways in Ape1- and/or Chk2-depleted cells, we measured homologous recombination (HR) and non-homologous end-joining (NHEJ) repair activities of these cells. We found that depletion of Ape1 caused around 50% reduction in HR repair activity in U2OS-DR cells^36^ (Fig. 3C). As a positive control, downregulation of BRCA1 reduced the HR-directed repair of the GFP reporter around 80%. In contrast, the additional depletions of Chk2 in Ape1-deficient cells revealed a slight, but significant recovery in the percentage of the HR repair compared to Ape1-deficiency alone, with either of two Ape1-specific siRNAs. The depletion of Chk2 alone did not alter the rate of HR repair (Fig. 3C). We also measured the NHEJ repair activities of these cells using a GFP-reporter assay system in H1299 human lung cancer cells^37^. As a control, the depletion of BRCA1 yielded the highest ratio of the green cells (Fig. 3D), indicating that (as expected) suppression of BRCA1 increases NHEJ. Similarly, but to a lesser extent, the depletion of Ape1 with two different Ape1-specific siRNAs caused a significant increase in the number of green cells relative to the control (Fig. 3D). These results suggest that deficiency of Ape1 supports NHEJ of DNA as a prevailing repair pathway. In contrast, the combined depletions of Ape1 and Chk2 resulted in a reduced rate of NHEJ repair of the GFP-reporter, relative to the single depletions of these proteins (Fig. 3D). Thus, our data suggest that Ape1 facilitates HR, and counteracts NHEJ of DNA ends.

We further examined whether the deficiency of Ape1 and/or Chk2 influences the recombination events between the sister chromatids. For this purpose, we quantified the ratio of the chromosomes with sister chromatid exchanges (SCE) in the Ape1- and/or Chk2-deficient U2OS cells, and found that the depletion of Ape1 alone had no effect on the frequency of SCE (Fig. 3E,F). However, the depletion of Chk2 alone led to a small increase in SCE, and the joint depletion of both proteins resulted in a ~7-fold increase in the number of chromosomes with SCE. As control, the cells depleted for BLM displayed the highest frequency of SCE (Fig. 3E,F). These data support our previous findings that once the Ape1/Chk2 signaling becomes limited, cancer cells preferably activate HR-directed repair.

### Damage site recruitment of BRCA1 and Rad51 are impaired in Ape1-depleted cells, and it is rescued by Chk2 depletion

**Figure 4:**
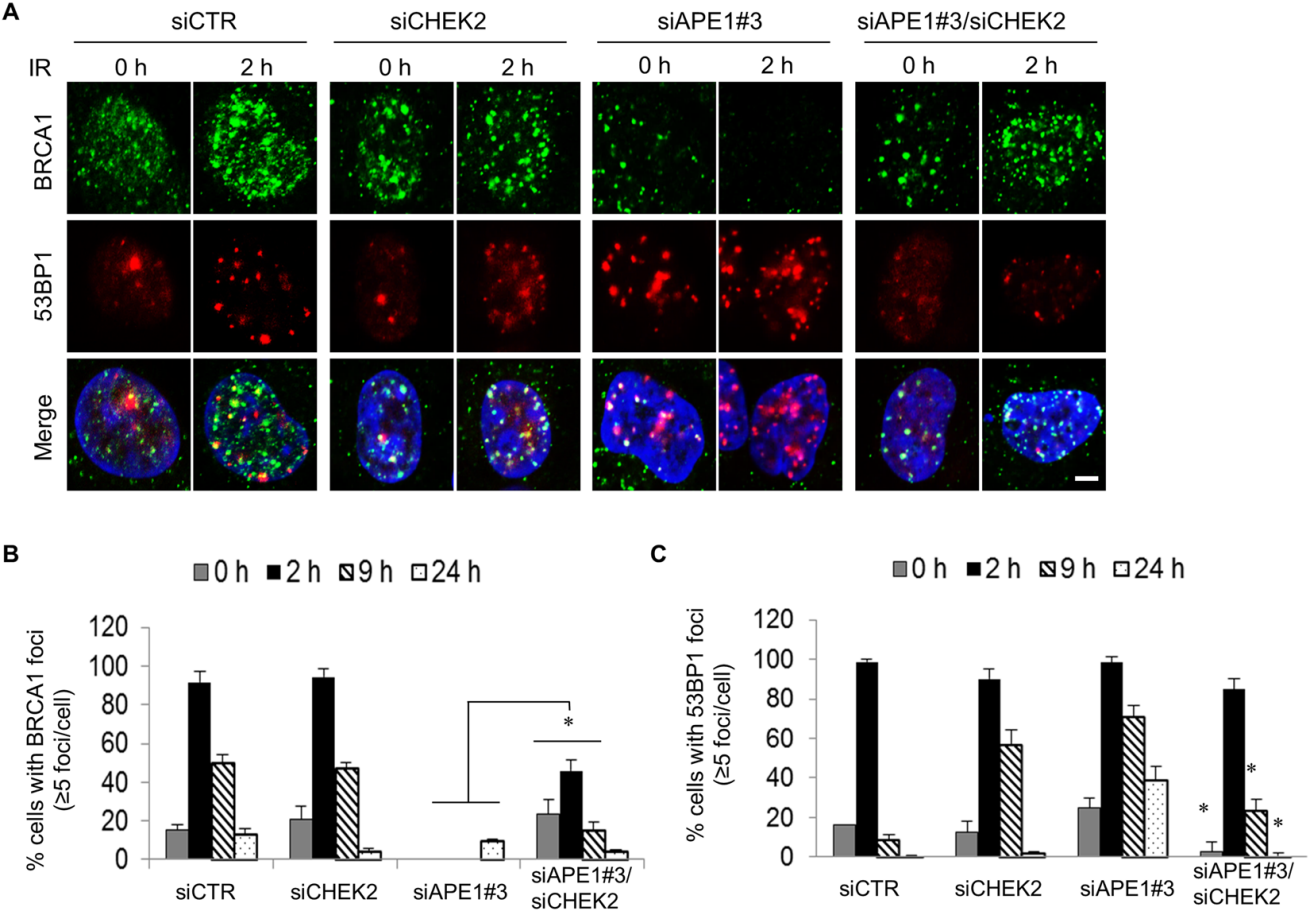
Impaired recruitment of BRCA1 to damage sites in Ape1-depleted cells, and its restoration by simultaneous depletion of Chk2. **(A)** U251-MG cells treated with the indicated siRNAs were exposed to IR (3 Gy), fixed at the indicated time points, and immunostained for BRCA1 and 53BP1. Scale bar, 5 µm. Cells with more than five BRCA1 (**B**) or 53BP1 foci (**C**) were quantified by counting at least 100 cells per sample. Error bars indicate standard deviations obtained from at least three independent experiments. *p < 0.01;Student’s *t* test.

**Figure 5:**
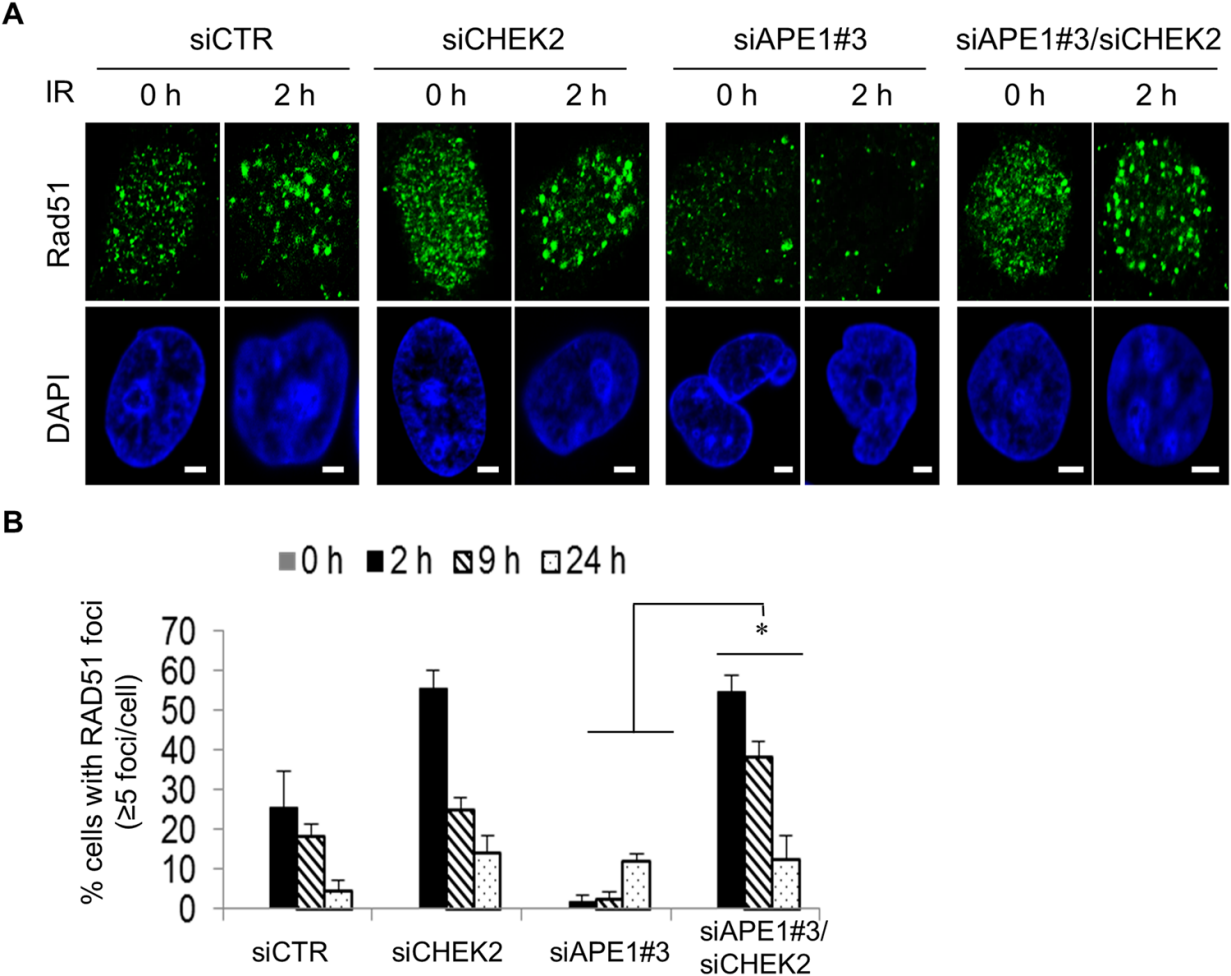
Impaired recruitment of Rad51 to damage sites in Ape1-depleted cells, and its restoration by simultaneous depletion of Chk2. **(A)** siRNA-treated U251-MG cells were exposed to IR (3 Gy), fixed at the indicated time points, and immunostained for Rad51. Scale bars, 5 µm. (**B)** Rad51 foci were quantified as described in Fig. 4. *p < 0.05; (siAPE1#3 vs. siAPE1#3/siCHEK2); Student’s *t* test.

Following the idea that Ape1 plays a role in DSB repair of DNA, we visualized the chromatin recruitment of 53BP1 and BRCA1, which facilitate NHEJ or HR, respectively, in U251-MG cells after an exposure to IR. Our findings revealed that depletion of Ape1 in U251-MG cells caused formation of large 53BP1 aggregates, which was accompanied by the impaired recruitment of BRCA1 to the damage sites (Fig. 4A,B). Consistently, the simultaneous depletion of Chk2 greatly reduced the formation of 53BP1 aggregates in Ape1-depleted cells, and that was coincided with the restored BRCA1 recruitment to the damage sites (Fig. 4A–C). Furthermore, the damage site recruitment of Rad51, a key mediator of HR, was also impaired in Ape1-depleted cells, which was subsequently restored by the depletion of Chk2 (Fig. 5A,B). Taken together, these findings indicate that Ape1, in coordination with Chk2, plays a critical role in DNA repair pathway choice by regulating the damage site recruitments of DSB DNA repair protein complexes. While no transcriptional upregulation of *BRCA1* and *RAD51* (Fig. S6F–H), or no significant alteration in the cell cycle distribution of Ape1 and/or Chk2-depleted cells after IR or TMZ (Fig. S7) was detected, the data suggest that increased protein stability and damage site recruitment of BRCA1 and Rad51 may play a role in the enhanced HR activity of the Ape1/Chk2-deficient cells.

### Coordinated regulation of Ape1 and Chk2 plays a critical role in glioblastoma response to TMZ

Next, we investigated whether TMZ tolerance of glioblastoma cells is linked to the HR activity that is regulated by the Ape1/Chk2 signaling. The analyses of twenty glioblastoma tumor tissues showed a positive correlation between Ape1 and Chk2 both at the protein (r = 0.65; p = 0.0015; n = 20) and mRNA (r = 0.61; p = 0.0037; n = 20) levels (Fig. S8A–D). A similar trend was also obtained from the primary cell cultures established from another set of glioblastoma specimens (n = 23) (Fig. S8E–H). In contrast, no significant correlation was detected between MGMT and Ape1 or Chk2 in the same tissues and cell cultures (Fig. S9A–D). These data indicate that Ape1 and Chk2 are coordinately regulated in glioblastoma tumor cells.

**Figure 6:**
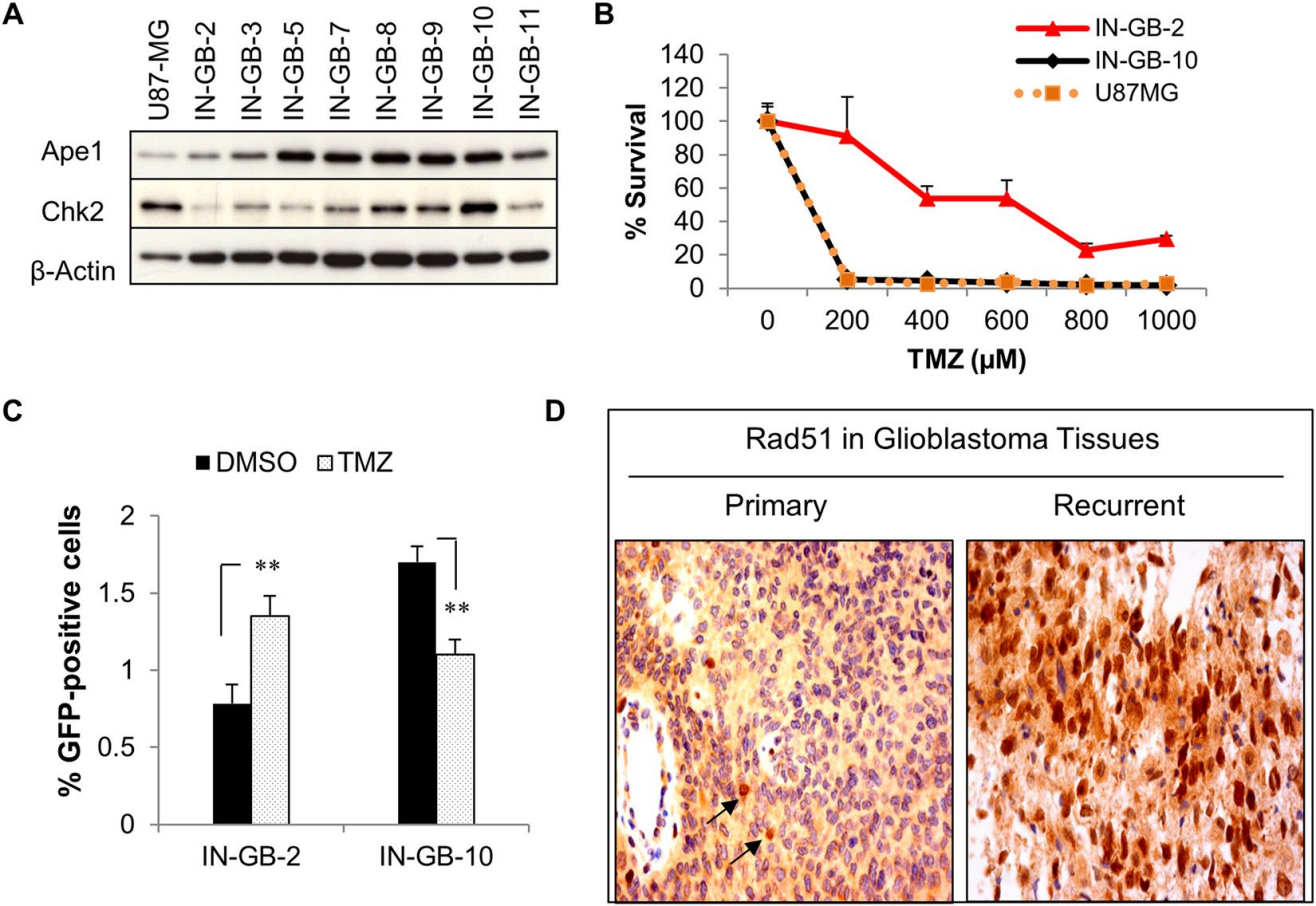
Glioblastoma resistance to TMZ is associated with HR activity that is regulated by cooperation of Ape1 and Chk2. **(A)** Western blot analyses of patient-derived early passage glioblastoma cells for Ape1, Chk2, and β-Actin. U87-MG served as internal control. **(B)** Patient-derived cells were treated with the indicated concentrations of TMZ for 1 h, and incubated for 10 days following extensive washing. Cells were counted, and percentage of survivals was calculated. **(C)** IN-GB-2 and IN-GB-10 cells were transfected with DR-GFP reporter gene and an I-Sce I plasmid. After 48 h, cells were treated with TMZ (1000 µM) for 1 h, and incubated for 24 h. GFP-positive cells were quantified by flow cytometry. Percentage of GFP-positive cells is shown. **p < 0.001; Student’s *t* test. **(D)** Primary and recurrent tissue samples of a glioblastoma patient were stained with Rad51 antibody. Arrows indicate Rad51-positive nuclei (brown).

Addressing whether the coordinated expressions of Ape1 and Chk2 is associated with TMZ response of glioblastoma cells, we analyzed two patient-derived early-passage glioblastoma cells IN-GB-2 and IN-GB-10 that are characterized by differential levels of Ape1 and Chk2 expressions, both at the protein and mRNA level (Fig. 6A; Fig. S8E,G). Analyses of the TMZ responses of these cells revealed that IN-GB-2 cells with lower Ape1/Chk2 levels were more resistant to TMZ than were in IN-GB-10 cells with their higher Ape1/Chk2 levels (Fig. 6B). Furthermore, following an exposure to TMZ, we detected an increased HR activity in IN-GB-2 cells, while IN-GB-10 cells showed a marked reduction in HR upon TMZ exposure (Fig. 6C). TMZ- or IR-induced nuclear accumulation of BRCA1 in IN-GB-2 cells, but not in IN-GB-10 cells, provided further evidence for the enhanced HR activity of the TMZ-resistant IN-GB-2 glioblastoma cells (Fig. S10A,B). These results suggest that Ape1/Chk2 signaling plays a critical role in TMZ response of glioblastoma cells, most likely by facilitating HR-directed repair that enables cellular adaptation to DNA alkylation.

We then postulated that suppression of Ape1/Chk2 signaling might be one of the defense mechanisms of glioblastoma cells against chronic genomic challenges, which is associated with adoption of alternative ways of HR protein recruitment to damage sites. To test this idea, we isolated TMZ-resistant glioblastoma cells that are able to propagate in substantial amounts of TMZ (320 µM), using U251-MG cells and patient-derived IN-GB-10 cells (Fig. S10C,D). Immunofluorescence analyses of these cells showed that nuclear recruitments of Ape1 and Chk2 were impaired in the TMZ-resistant clones of these cells (Fig. S11A,B), while there was no significant change in the gene expressions of *APEX1* and *CHEK2* (Fig. S11C), or in the protein levels of Ape1 and Chk2 (Fig. S11D,E). In addition, BRCA1 was highly enriched in the nuclei of the TMZ-resistant clone of U251-MG cells (U251-MG-R) (Fig. S11F), as well as in two different clones of TMZ-resistant IN-GB-10 cells (IN-GB-10-R1 and IN-GB-R2) (Fig. S11G), while no significant change was detected in the expression of the *BRCA1*, *RAD51*, and *53BP1*genes in these cells (Fig. S11H).

Next, to investigate whether increased HR activity is associated with the recurrence of glioblastoma, we analyzed 38 matched glioblastoma tumor pairs (the term of “pair” represents primary and recurrent tissue samples obtained from the same patient) for the nuclear staining of Rad51 protein, as a marker of HR activity. Around 42% of the recurrent glioblastoma tissues (16 of 38) were scored as positive for the enrichment of Rad51 in nuclei, and a representative image from a patient is shown in Fig. 6D. Since all the patients diagnosed with primary glioblastoma received TMZ therapy prior to tumor recurrence, these results suggest that glioblastoma cells rapidly develop a defense system against TMZ by activating an alternative homology-directed recombination repair system.

**Figure 7:**
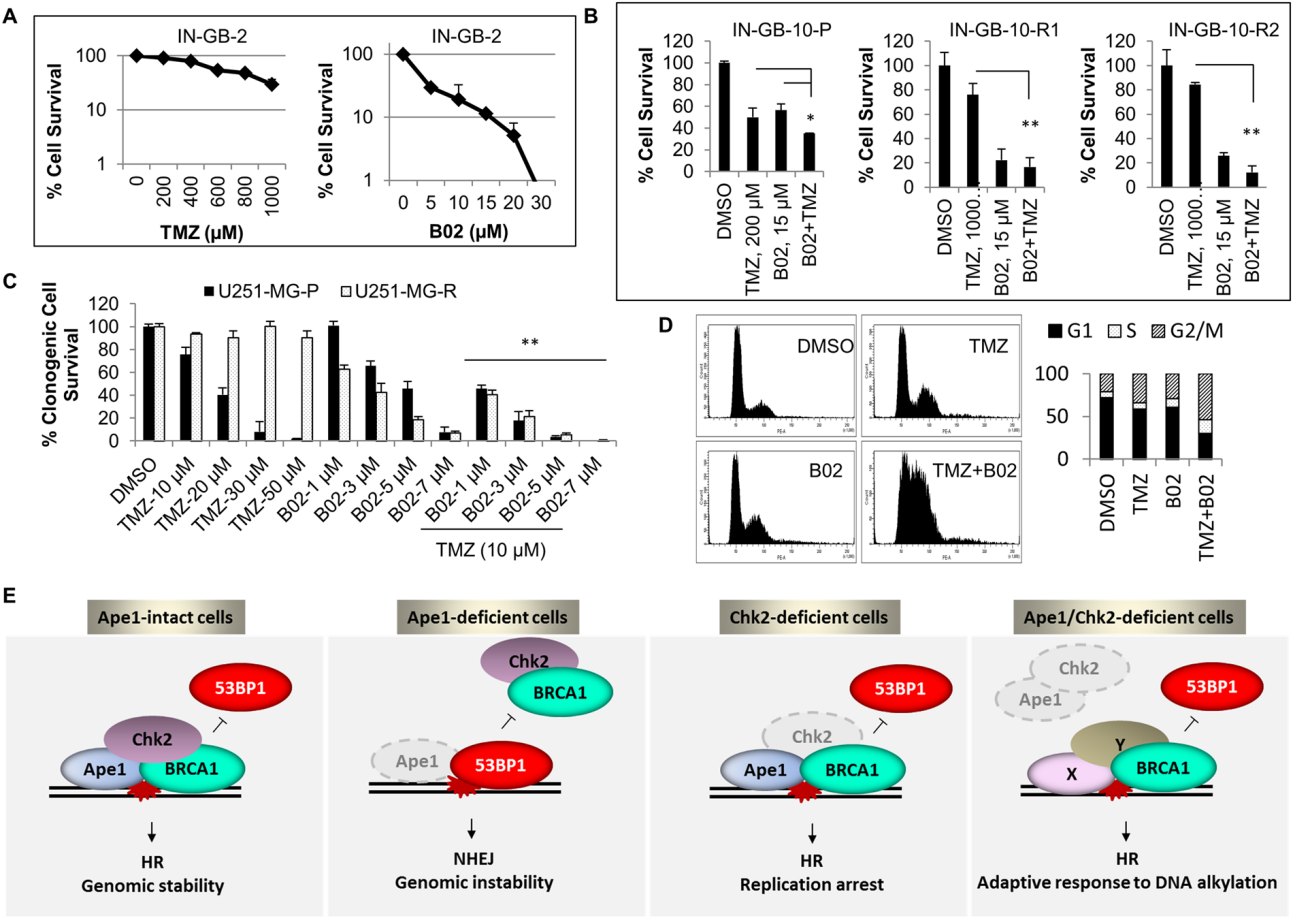
Interference of HR through a chemical inhibitor of Rad51 causes substantial toxicity in TMZ-resistant glioblastoma cells. **(A)** Intrinsically TMZ-resistant patient-derived IN-GB-2 cells were exposed to the indicated concentrations of TMZ or B02. After six days of incubation, cells were counted and cell survival was assessed by cell counting. Percentages of surviving cells were calculated in proportion to the cells treated with the vehicle (DMSO). **(B)** Parental TMZ-sensitive IN-GB-10 cells were exposed to increasing concentration of TMZ (10–320 µM) for three months. Two different clones (IN-GB-10-R1 and IN-GB-10-R2) growing in the presence of 320 µM of TMZ were isolated. Cells were treated with the indicated concentrations of TMZ and/or B02, and percentages of survival were calculated as described in A. *p < 0.1; **p < 0.001; Student’s* t* test. **(C)** U251-MG cells were exposed to increasing concentration of TMZ (10–320 µM) in a period of three months. One clone propagating in 320 µM of TMZ was isolated and cultured in the presence of TMZ for at least a month prior to the study. The parental (U251-MG-P) and resistant cells (U251-MG-R) were exposed to TMZ, B02, or combinations of TMZ and B02 at the indicated concentrations, and clonogenic cell survival assays were performed. Colonies were counted after seven days, and the percentage of survival was calculated in proportion to the control cells. **p < 0.05; Student’s﻿ *t﻿* test (combination versus single treatments). **(D)** U251-MG cells were exposed to TMZ (200 µM), B02 (20 µM), or the combination of TMZ (200 µM) and B02 (20 µM) for 24 h. The representative histograms of the cell cycle analyses and quantification of the cell cycle stages are shown. **(E)** Intact Ape1/Chk2 signaling is required for recruitment of key HR proteins such as BRCA1 and Rad51 to facilitate HR, possibly through Ape1-mediated 3′ DNA-end processing of double-strand breaks. When Ape1 is deficient, simple base lesions are converted to complex clustered DNA lesions, which is further augmented after exposure to DNA DSB damages. Any possible failure in Ape1-mediated DNA-end processing results in impaired BRCA1 recruitment to damage sites, by which error-prone NHEJ becomes predominant repair pathway in Ape1-deficient cells, causing chromosome fusions and cell death. While deficiency of Chk2 causes replication defects and cell death, shortage of Ape1 and Chk2 is likely to create a new platform for alternative DNA end-processing factors that facilitate recruitment of HR proteins, increasing HR activity and thereby adaptation to constant genomic challenges.

Finally, we asked whether HR-“addicted” drug-resistant glioblastoma cells can be specifically targeted by inhibition of HR. To block the activity of HR, we used a chemical inhibitor of Rad51, B02^13^. We observed that B02 had a greater efficiency on the patient-derived IN-GB-2 cells that are intrinsically resistant to TMZ, compared to its efficiency on the TMZ-sensitive patient-derived IN-GB-10 cells (Fig. 7A,B). In addition, B02 proved to be more effective on two different TMZ-resistant clones of IN-GB-10 cells (Fig. 7B). Similarly, the effect of B02 was rather potentiated in the TMZ-resistant clone U251-MG-R cells, compared to its parental counterpart (Fig. 7C). Moreover, a synergistic cell killing effect was observed when B02 was combined with TMZ both in IN-GB-2 and U251-MG cells (Fig. 7B,C). The cell cycle analyses of U251-MG cells demonstrated that the combination of B02 with TMZ leads to a strong cell cycle arrest at the G2/S phase of cell cycle (Fig. 7D). These data confirm the HR-dependency of the glioblastoma cells that are refractory to TMZ therapy, and also demonstrate the higher efficiency of B02 in HR-“addicted” glioblastoma cells.

## Discussion

Ape1-mediated incision of AP sites is an essential step for the repair of DNA lesions generated during routine cellular metabolism^38^. Here, we show that, in addition to its direct involvement in base-excision repair of DNA, Ape1 also participates in the coordination of DSB repair of DNA. While Ape1 directs correct repair of simple base damages of DNA through BER, it counteracts error-prone processing of DNA lesions via non-homologous end joining repair pathway, on the contrary facilitating HR-directed repair. Importantly, intact Chk2 signaling appears to be necessary for proper channeling of the repair pathways to ensure genome stability.

Given that Ape1 is rapidly recruited to the sites of DSBs (Fig. 2A,B), Ape1 may function in the processing of DNA ends. Because Ape1 possesses enzymatic activities such as 3′-5′ exonuclease, 3′-phosphatase, and 3′-phosphodiesterase, the repair of clustered DNA lesions may require Ape1 activities to process 3’-end blocking groups during DSB repair of DNA.

Our studies indicate that intact Ape1/Chk2 signaling is required for correct repair of base damages via BER, ensuring strong checkpoint control and genomic stability. In Ape1 deficiency, simple base lesions cannot be accurately repaired, and AP sites are formed. Due to the checkpoint defects, Ape1-deficient cells may undergo DNA replication that possibly induces formation of complex clustered DNA lesions consequential to the accumulation of AP sites^39, 40^. Following induction of such complex DNA lesions by IR, NHEJ appeared to be the predominant repair pathway in Ape1-depleted cells. Consistent with these findings, we recently reported that shortage of Ape1 causes severe telomere defects characterized by telomere fusions^22^. Thus, genomic instability observed in Ape1-defective cells might be originated, at least in part, from the mutagenic repair caused by provoked activity of error-prone NHEJ.

Improved cell survival following dual depletion of Ape1 and Chk2 in glioblastoma cells encouraged us to hypothesize that Ape1/Chk2 signaling coordinates DNA repair pathways to ensure efficient repair of DNA. Accordingly, our findings showing that HR is restored in Ape1/Chk2-deficient cells point toward an adaptive response of glioblastoma cells to constant genomic stress. In addition, the possible switch between DNA repair pathways may be directed by Ape1-parallel pathway(s) which subsequently becomes complementary in response to lack of Ape1 activity. A possible scenario can be envisioned for TDP1 (Tyrosyl-DNA phosphodiesterase 1) that has been implicated in processing of 3′-blocking lesions of DNA^41, 42^. In the absence of Ape1, combined with a checkpoint defect (Chk2 deficiency), processing of 3’-lesions by other factors such as TDP1 might cause a switch between DNA repair pathways.

Finally, our study identifies a previously unknown unique role for Ape1 in the participation of different DNA repairs. Based on our model (Fig. 7E), Ape1 facilitates HR, most likely participating in DNA-end processing of complex damages. Pro-HR activity of Ape1 counteracts NHEJ on the break sites, which prevents error-prone end joining of DNA ends. DNA damage checkpoint kinase Chk2 appears to be a regulatory factor for Ape1 in this process, likely through its kinase activity and/or its influence on the stability of the associating protein complexes. Intriguingly, chromosome fusions and cytotoxicity caused by Ape1 deficiency can be counteracted by Chk2 depletion, and that promotes HR activity. It is likely that dual deficiency of Ape1 and Chk2 creates a new platform for the establishment of an adaptive alternative repair system that is orderly engaged by the cell to establish a defense system against continuous genomic stress to DNA. Supporting this idea, our study provides evidence for the functional significance of the Ape1/Chk2 connection in glioblastoma chemotherapy, and proposes a mechanism of resistance to the chemotherapeutic agent TMZ through Ape1/Chk2-regulated DNA repair activities. However, detailed role(s) of Ape1 in homology-directed recombinational repair and its mode(s) of regulation by Chk2 kinase need further investigation.

In summary, we discovered that Ape1 facilitates HR, and counteracts NHEJ on double-strand break sites of DNA. Using glioblastoma tumor model, we also showed that the regulatory protein complex of Ape1 and Chk2 plays a critical role in the course of adaptation to continuous DNA alkylation, and contributes to the choice of DNA repair program. Taking advantage of the “HR addiction” of TMZ-resistant glioblastoma cells, we also demonstrated that inhibition of Rad51, in combination with TMZ, might be a promising strategy for glioblastoma treatment. Thus, our study lightens up molecular insights of TMZ resistance, and offers a mechanism-based alternative therapy for primary and recurrent glioblastoma patients.

## Methods

### Transient knockdown of proteins

Ape1 was transiently knocked down in cells using three different synthetic siRNAs: APE1-siRNA#1 (siAPE1#1), 5′- GUC UGG UAC GAC UGG AGU A-3′ (Qiagen); APE1-siRNA#2 (siAPE1#2), 5′-CCU GCC ACA CTC AAG AUC U-3′ (Qiagen), and APE1-siRNA#3 (siAPE1#3), (Invitrogen, HSS100557). Two different CHEK2 siRNAs; CHEK2-siRNA#1 (Santa Cruz, sc-29271) and CHEK2-siRNA (Invitrogen, VHS40242) were used in the study. BRCA1-siRNA (HSS186096), RAD51-siRNA (VHS40453) and control-siRNA (12935–300) were purchased from Invitrogen. Cells were transfected with siRNAs by using Lipofectamine-2000 (Invitrogen) according to the manufacturer’s instructions at the final concentration of 50 nM.

### Immunofluorescence

Cells were grown on coverslips, and fixed with methanol after the indicated treatments. Following blocking with 3% BSA for 1 h, slides were incubated with the primary antibodies at 4 °C for overnight, washed three times, and incubated with the secondary antibodies (Alexa Fluor 488 goat anti-mouse IgG or Alexa Fluor 594 donkey anti-rabbit IgG, 1:700 dilution) at room temperature for 1 h. Slides were mounted on Vectashild-DAPI mounting medium (Vector Laboratories). Primary antibodies used for immunofluorescence studies were anti-Ape1 (Santa Cruz, sc-17774, 1:200), anti-phospho-Chk2 (Thr68) (Cell Signaling, #2661, 1:200), anti-BRCA1 (Calbiochem, MS110, 1:100), anti-γH2AX (Cell Signaling, #9718, 1:500), anti-53BP1 (Cell Signaling, #4937, 1:100), and anti-Rad51 (Abcam, ab213, 1:200). Images were taken by confocal laser scanning microscopy (LSM780, Zeiss), and analyzed by ZEN software (Zeiss).

### Microirradiation

Cells were exposed to laser microirradiation by using an inverse spinning disc confocal microscope unit, equipped with CW lasers (405, 488, 561 or 640 nm laser) and a pulsed 355 nm laser diode. Live-imaging was performed by using 488 nm laser (GFP) and 561 nm laser (RFP) for 30 min as described in the supplementary experimental procedures. Absolute fluorescence intensities of the laser-microirradiated areas were calculated by subtracting the cellular background fluorescence from the laser-induced damage site fluorescence for each time-point (Image J, NIH). Error bars represent standard deviation of three independent measurements.

### Immunohistochemistry

Immunohistochemistry was performed on formalin-fixed, paraffin-embedded (FFPE) tumor tissues as described in the supplementary experimental procedures.

### Cell cycle analysis

For cell cycle analysis, ethanol-fixed cells were stained with propidium iodide and analyzed for DNA content by flow cytometry (BD FACS Canto II). Data were analyzed using BD FACS Diva Software v6.1.2.

### Tissue samples

Tissue samples were obtained from patients with newly diagnosed glioblastomas. All human samples were collected according to the Institutional Review Board protocols approved by the Medical University of Vienna. Informed patient consents had been obtained for human tissue samples in the study. Histological diagnosis was performed on paraffin embedded specimens according to the World Health Organization (WHO) criteria^43^. As non-neoplastic brain tissue, normal appearing white matter specimens (NWM) were obtained from patients who had undergone surgery for epilepsy. Snap-frozen tissue samples were homogenized for Western blotting and PCR analysis.

### Patient-Derived Early Passage Glioblastoma Cells

Tumor samples were minced, treated with trypsin for 1 h, and cultured in DMEM supplemented with 10% fetal bovine serum, 2% L-glutamine (27 mg/ml), 1 x non-essential amino acids and 1% penicillin/streptomycin (100 IU/ml penicillin, 100 µg/ml streptomycin) at 37 °C in 5% CO2. The tumor cell cultures thus generated have been cryopreserved at low passages and propagated for at least 4 months. TP53 sequencing revealed that IN-GB-2 has wild-type TP53, whereas IN-GB-10 has a mutation in codon 216 (c.646G > A). PTEN sequencing showed that IN-GB-2 as well as IN-GB-10 have PTEN mutations in codon 319/320 (c.955_958delACTT) and codon 83 (c.249C > A), respectively. All mutations were confirmed in the corresponding tissue samples.

### Western Blot, Co-immunoprecipitation Studies, and Chromatin isolation

Whole cell and tissue lysates were prepared in lysis buffer (10 mM Tris-HCl pH 7.4, 150 mM NaCl, 5 mM EDTA, pH 8.0, 1% Triton X-100) containing protease inhibitors (Roche). Protein concentrations were determined by Bradford assay (Bio-Rad), and samples containing equivalent amounts of protein (20–30 µg) were subjected to SDS-PAGE. Immunoblotting was carried out as described in Supplemental experimental procedures. For co-immunoprecipitation studies, HEK293 cells were treated with BSA (0.5%; 24 h), double thymidine (DT, 2 mM), hydroxyurea (HU, 2 mM; 16 h), or methyl methane sulphonate (MMS, 250 µM; 4 h), along with asynchronized (Asyn.) cells. MMS-treated cells were washed with PBS several times, and incubated for another 12 h before harvesting. In another set of co-immunoprecipitation reactions, HEK293 cells were treated with temozolomide (TMZ, 250 µg/ml; 4 h) or exposed to ionizing radiation (IR, 3 Gy). TMZ-treated cells were washed with PBS several times, and incubated at 37 °C for another 4 h prior to the harvest. Following the IR exposure, cells were immediately returned to the incubator and harvested 6 h after the IR exposure. Cells were then subjected to immunoprecipitation (IP) with a Chk2 antibody, as described in the Supplemental Experimental Procedures. Chromatin isolation was performed as described earlier^44^.

### Preparation of Metaphase Chromosomes

RNAi-treated U251-MG cells were treated with DMSO (control) or TMZ (1000 µM, 1 h) at 48 h of siRNA transfection. After extensive washing, cells were treated with nocodazole (Sigma) (100 ng/ml) for 16 h, and mitotic cells were harvested by mitotic shake-off. Metaphase chromosomes were prepared according to the standard protocol as described in Supplemental Experimental Procedures.

### Frequency of Sister Chromatid Exchange (SCE)

Frequency of SCE was determined in Ape1 and/or Chk2-downregulated U2OS cells as described previously^45^, with small modifications, as described in Supplemental Experimental Procedures.

### Cell Viability Assays

Glioblastoma cells were seeded in 6-well plates at a density of 30,000 cells per well. Next day, cells were treated with vehicle (DMSO) or the indicated concentrations of TMZ for 1 h. After an extensive wash, cells were cultured for 6–10 days, and harvested with trypsinization. After staining with trypan blue (0.4%, Invitrogen), cells were counted by using a Neubauer cell counting chamber. Percentage of survival is represented as mean percent relative to the vehicle-treated cells ± SD of three independent experiments. Significance between the groups was analyzed by Student’s *t* test.

### Clonogenic Survival Assay

48 h after siRNA transfections, U251-MG cells were incubated with the indicated concentrations of temozolomide for 1 h. Cells were then seeded (500 cells/35 mm plates), and cultured for 7 days, followed by staining with Giemsa solution. Surviving colonies consisting of more than 50 cells were counted, and percentage of survival was expressed as ratio of survival against that of DMSO-treated cells (control). The data were obtained from three independent treatments, and p values (siAPE1#3 vs. siAPE1#3/siCHEK2) were calculated by Student’s *t* test. *p < 0.05.

### HR and NHEJ Assay

HR assay^36^ and NHEJ assay^37^ were previously described. Detailed experimental procedures can be found in the supplementary experimental procedures.

## Electronic supplementary material

Supplementary Info
