# Supplementary material for: Ape1 guides DNA repair pathway choice that is associated with drug tolerance in glioblastoma
Source: Sci Rep. 2017 Aug 29;7:9674. doi: 10.1038/s41598-017-10013-w (PMC5574897; doi:10.1038/s41598-017-10013-w)
Supplement: Supplementary file 1 — Supplementary Info [file 41598_2017_10013_MOESM1_ESM.pdf]

Ape1 guides DNA repair pathway choice that is associated with drug tolerance in glioblastoma

Thomas Ströbel[1], Sibylle Madlener[2], Serkan Tuna[2], Sarah Vose[3], Tonny Lagerweij[4], Thomas Wurdinger[4,5], Klemens Vierlinger[6], Adelheid Wöhrer[1], Brendan D. Price[7], Bruce Demple[8], Okay Saydam[2,5] and Nurten Saydam[2,5,*]

[1]Institute of Neurology, Medical University of Vienna, A-1090 Vienna, Austria

[2]Molecular Neuro-Oncology Research Unit, Department of Pediatrics & Adolescent Medicine, Medical University of Vienna, A-1090 Vienna, Austria

[3]Vermont Department of Public Health, 108 Cherry St., Burlington, VT 05402, USA

[4]Neuro-Oncology Research Group, Department of Neurosurgery, VU University Medical Center, Amsterdam, 1081 HV Amsterdam, The Netherlands

[5]Department of Neurology, Massachusetts General Hospital, Harvard Medical School, Charlestown, MA 02129, USA

[6]Molecular Diagnostics, AIT - Austrian Institute of Technology, A-1190 Vienna, Austria

[7]Department of Radiation Oncology, Division of Genomic Instability and DNA Repair, Dana-Farber Cancer Institute, Harvard Medical School, Boston, MA 02115, USA

[8]Department of Pharmacological Sciences, Stony Brook University, School of Medicine, Stony Brook, NY 11794-8651, USA

[*]Corresponding Author

SUPPLEMENTAL FIGURE LEGENDS

Figure S1, related to Figure 1A and B

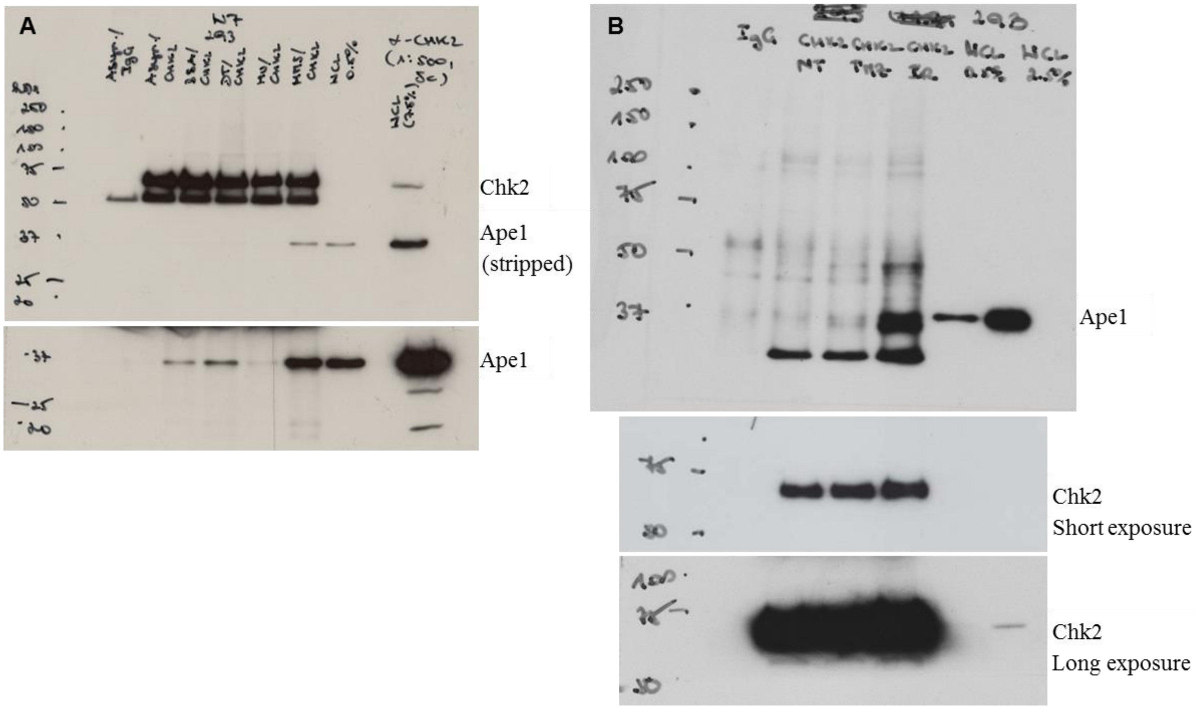

Figure S1. (A) Original membranes of Chk2 and Ape1 immunoblot following immunoprecipitation with a Chk2 antibody. The membrane was incubated first with an Ape1 antibody, then stripped and reblotted with a Chk2 antibody. Relatively weaker Ape1 signal represents the remaining signal after membrane stripping. **(B)** Original membrane of Ape1 and Chk2 immunoblot following immunoprecipitation with a Chk2 antibody after the exposure to temozolomide (TMZ) or ionizing radiation (IR). Due to the weak Chk2 signal in the input (2.5% of the IP reaction), the membrane was exposed for a longer time (long exposure).

Figure S2, related to Figure 1C and D

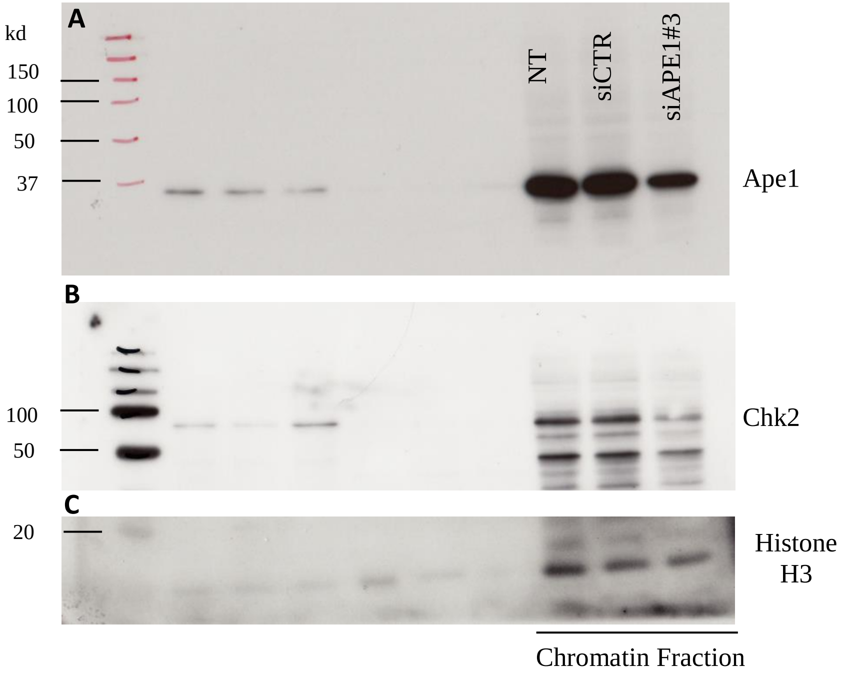

Figure S2. Chromatin fractions were isolated after 72 h of siRNA transfections, and subjected to Western blot. **(A)** Original membrane of Ape1 immunoblot **(B)** Chk2 immunoblot **(C)** Histone H3 immunoblot are shown.

Figure S3, related to Figure 1

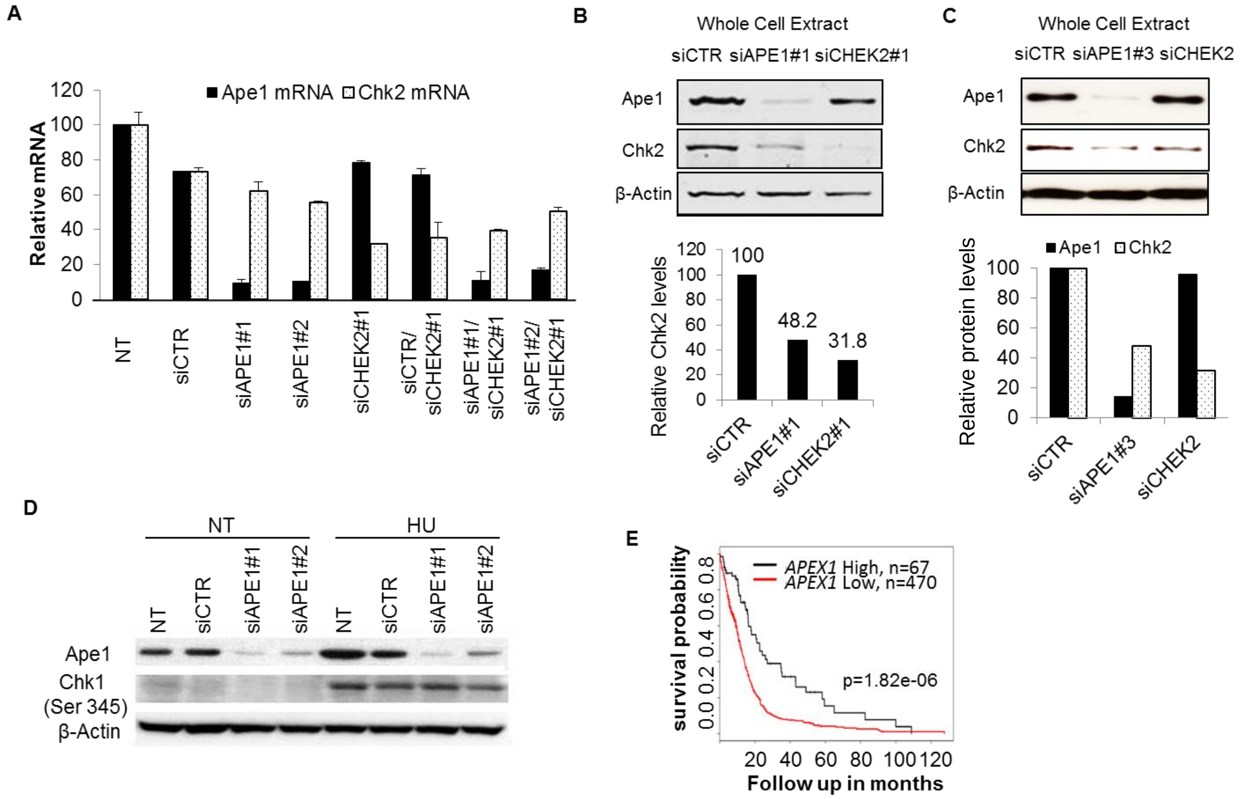

Figure S3. DNA damage checkpoint defects in Ape1-depleted cells (A) siRNA down-regulation of Ape1. U2OS cells were transfected with control siRNA (siCTR), two different APE1-siRNAs (siAPE1#1 and siAPE1#2), CHEK1-siRNA#1 (siCHEK2#1), or combination of siAPE1#1 and siCHEK2#1, and harvested at 72 h post-transfection. Relative mRNA levels of *APEX1* and *CHEK2* were determined by real-time PCR, and normalized to the *GAPDH* levels. **(B)** Chk2 levels are decreased in Ape1-depleted cells. U2OS cells were transfected with control-siRNA (siCTR), APE1-siRNA#1 (siAPE1#1), or CHEK2-siRNA#1 (siCHEK2#1), harvested at 72 h of transfection, and subjected to Western blotting. β-Actin was used as loading control. Quantification of data is presented in bars**. (C)** U2OS cells were treated with siCTR, APE1-siRNA#3 (siAPE1#3), or CHEK2-siRNA (siCHEK2), as in (B) and subjected to Western blot. Quantification of data is presented in bars**. (D)** Chk1 phosphorylation at serine-345 is not affected by Ape1 deficiency. U2OS cells were transfected with siCTR, siAPE1#1 or siAPE1#2. 72 h post-transfection, cells were treated with HU (1 mM) for 3 h. Ape1, phospho-Chk1 (Ser 345), and β-actin were detected by immunoblotting. **(E)** Increased expression of *APEX1* is associated with better survival of glioblastoma patients. Survival analysis of the glioblastoma patients for *APEX1* expression in the TCGA glioblastoma data set.

Figure S4, related to Figure 1

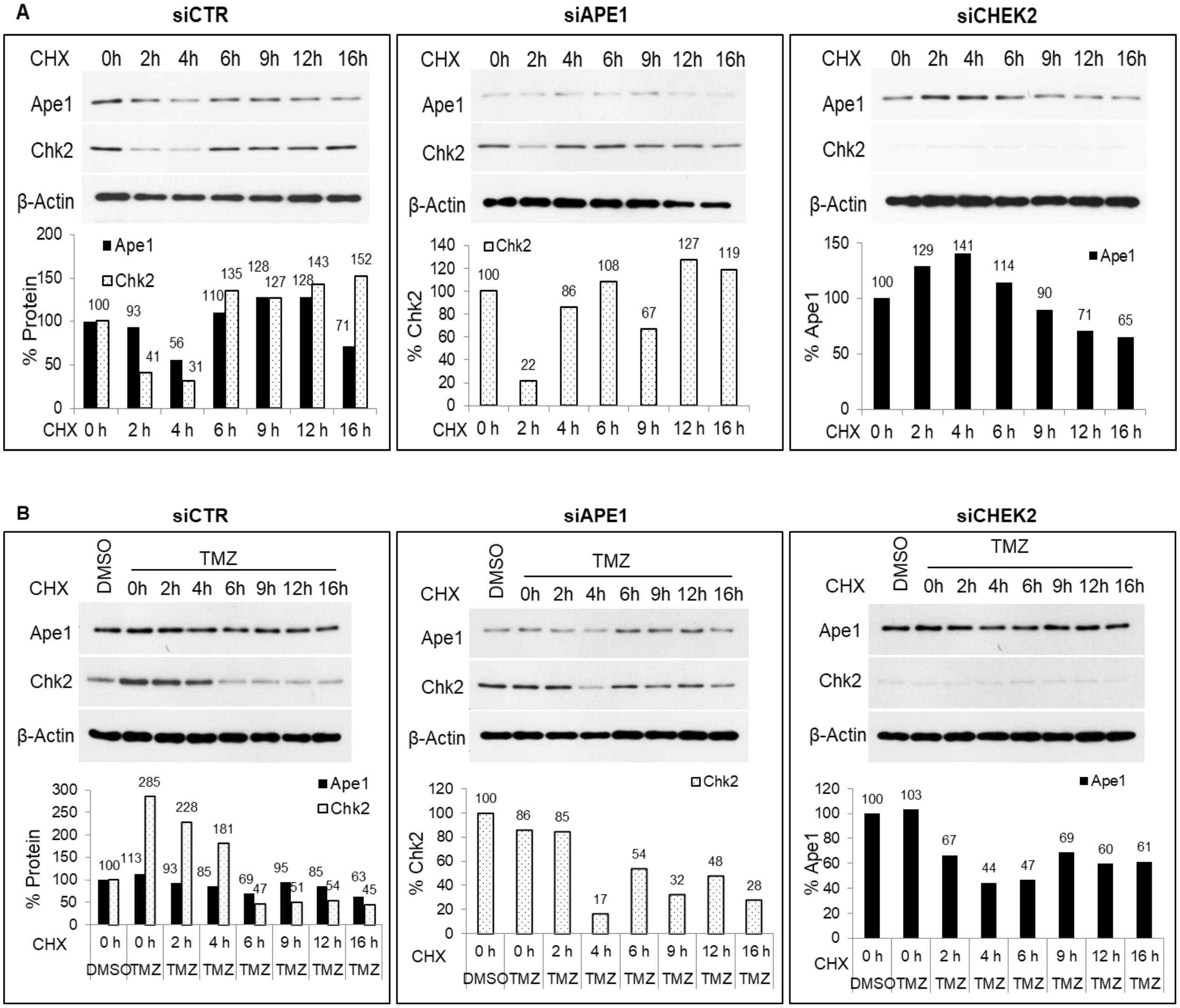

Figure S4. Protein half-life of Ape1 and Chk2 in U251-MG cells. (A) U251-MG cells were transfected with the indicated siRNAs. At 48 h of the transfection, cells were treated with cycloheximide (CHX, 50 µg/ml) for the indicated time points. Cells were then harvested and subjected to Western blot for Ape1 and Chk2 protein levels. β-Actin was used as loading control. **(B)** 48 h after siRNA transfection, U251-MG cells were treated with DMSO (control) or temozolomide (TMZ, 1000 µM) for 1 h. After several washings, cells were treated with CHX (50 µg/ml) for the indicated time points. Cells were harvested and analyzed by Western blot as in (A).

Figure S5, related to Figure 2

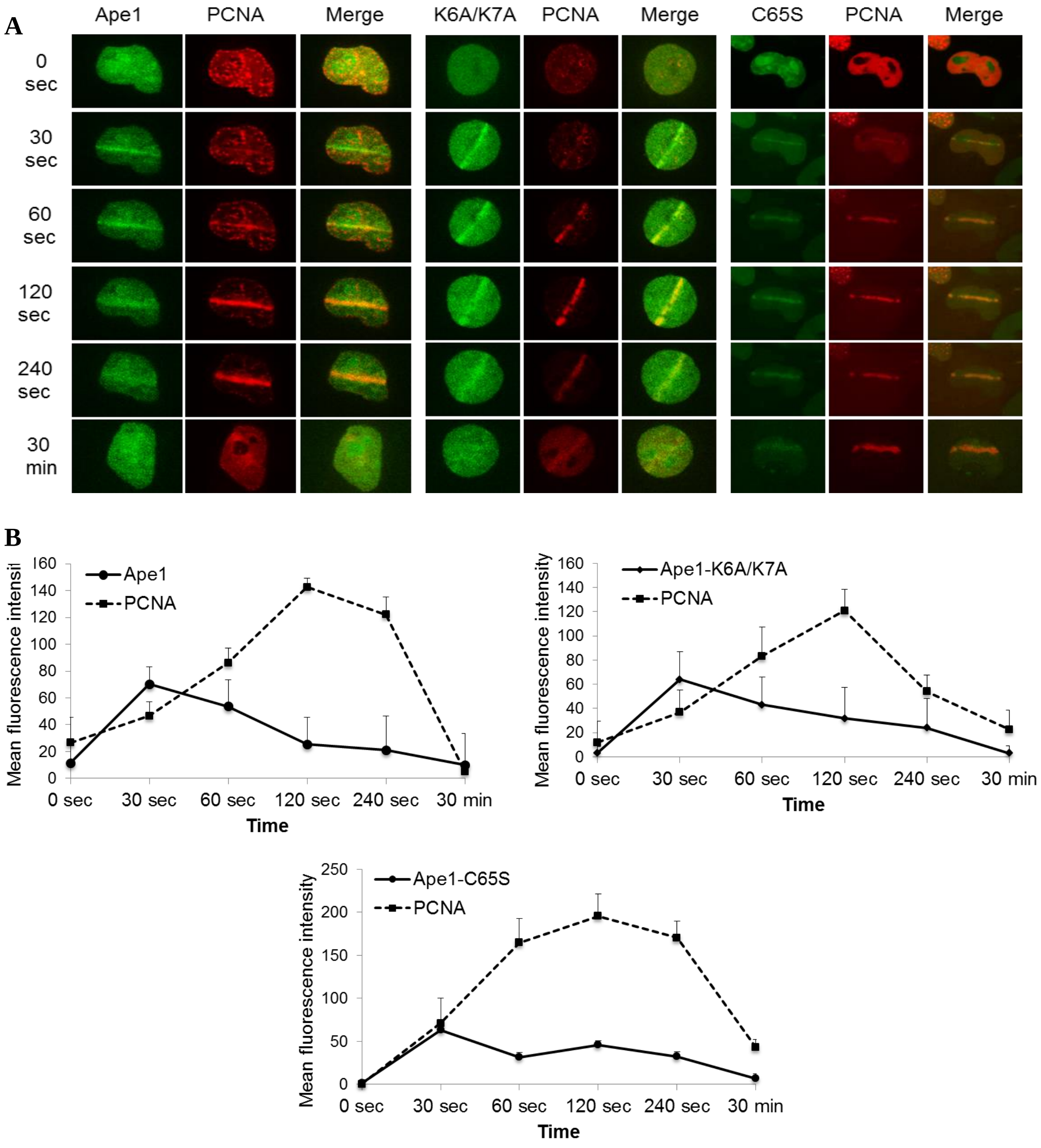

Figure S5. Recruitment of Ape1 to DSB sites is independent of its redox and acetylation-dependent gene regulation function. (A) U251-MG cells were infected with lentiviral particles carrying GFP-tagged wild-type, K6A/K7A mutant-, and C65S mutant-type of Ape1. After the G418 selection, cells were transfected with a RFP-PCNA plasmid. 48 h later, cells were exposed to laser micro-irradiation, and the cells showing green (Ape1) and red (PCNA) fluorescence were live-imaged for 30 min. At least three independent laser incisions were performed for each cell type expressing wild-type and mutant forms of Ape1. Representative images are shown. **(B)** Quantifications of the damage site recruitment of Ape1, Ape1-K6A/K7A, Ape1-C65S and PCNA are shown.

Figure S6, related to Figures 2-5

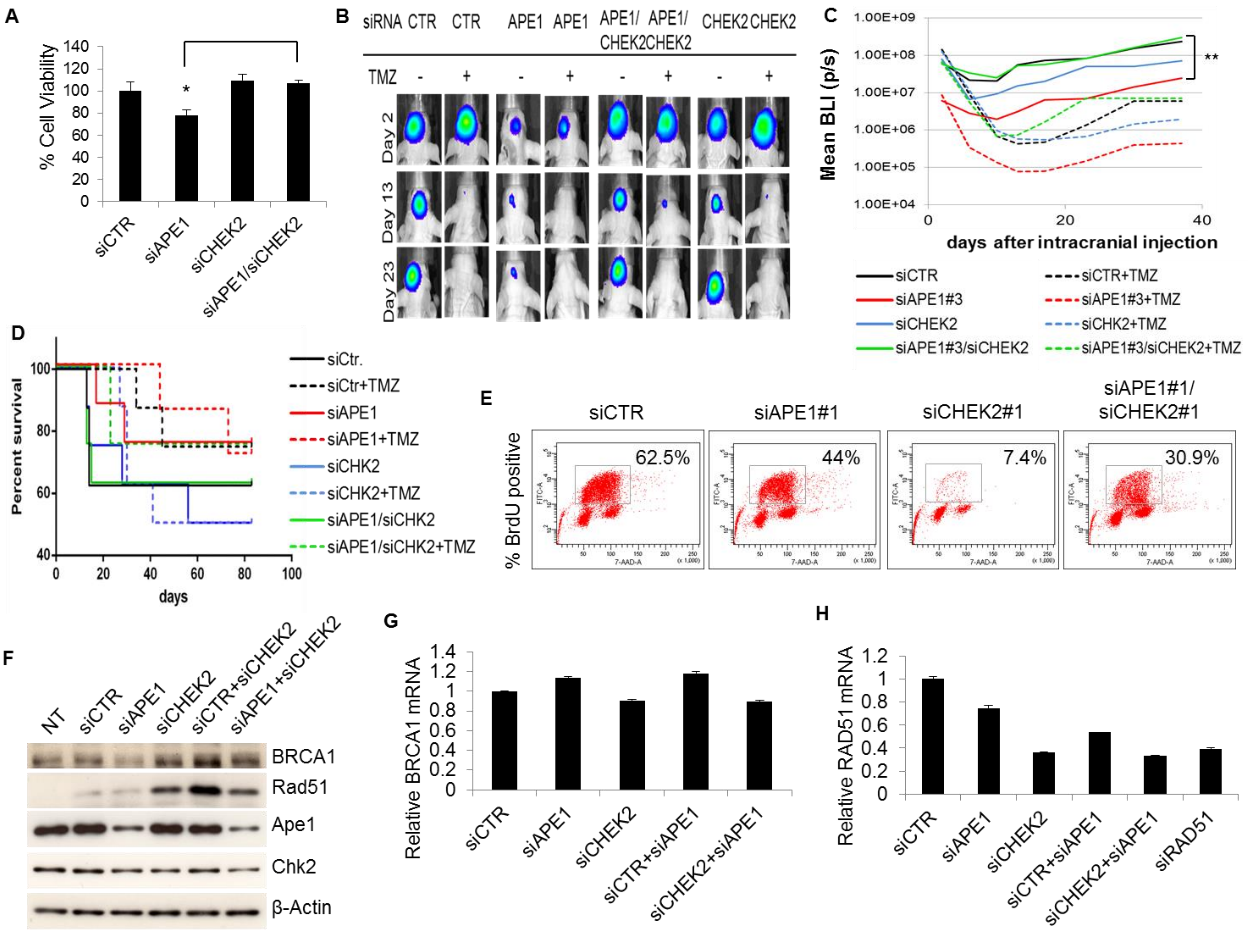

Figure S6. Combined deficiencies of Ape1 and Chk2 support glioblastoma tumor growth in mice. (A) U251-MG cells were transfected with the indicated siRNAs in triplicate. Cells were counted three days after the transfection, and percentage of cell viability was calculated by setting the control-siRNA treated sample as 100 %. *p<0.01; Student's *t* test. **(B)** Glioblastoma tumor growth in mice. U251-FM cells were transfected with the indicated siRNAs, and 16 h later injected in the striatum of mice (n=16 per group) via stereotaxic injection. TMZ (10 mg/kg) were intraperitoneally administered at day 2, day 3 and day 4 after intracranial injection of cells. Representative pictures from each group are shown. **(C)** Tumor growth was monitored by *in vivo* bioluminescence imaging (BLI) weekly. Photon counts per second (p/s) are given. **p=0.007 (siAPE1#3 vs. siAPE1#3/siCHEK2); Student's *t* test. **(D)** Kaplan-Meier survival curves are presented for each group. **(E)** Defective replication arrest of Ape1/Chk2-depleted cells. U2OS cells were transfected with control (siCTR), siAPE1#1, siCHEK2#1, or combination of siAPE1#1 and siCHEK2#1 siRNAs. 48 h later, cells were pulse-labeled with 10 µM BrdU for 40 min, and incorporation of BrdU was analyzed by flow cytometry. Histograms are representative of three independent experiments. Percentage of BrdU-positive cells is shown in graphs. **(F)** BRCA1 and Rad51 protein levels in Ape1 and/or Chk2-depleted cells. U251-MG cells were transfected with siCTR, siAPE1#3, siCHEK2, or with the combination of siAPE1#3 and siCHEK2. 72 h after the transfection, cell extracts were prepared, and cellular BRCA1, Rad51, Ape1, and Chk2 proteins were detected by immunoblotting. β-Actin was used as loading control. Total RNAs were also isolated after 72 h of transfection. *BRCA1* (**G**) and *RAD51* gene expressions (**H**) were analyzed by real-time PCR. The normalized values to *GAPDH* are shown.

Figure S7, related to Figures 4 and 5

A

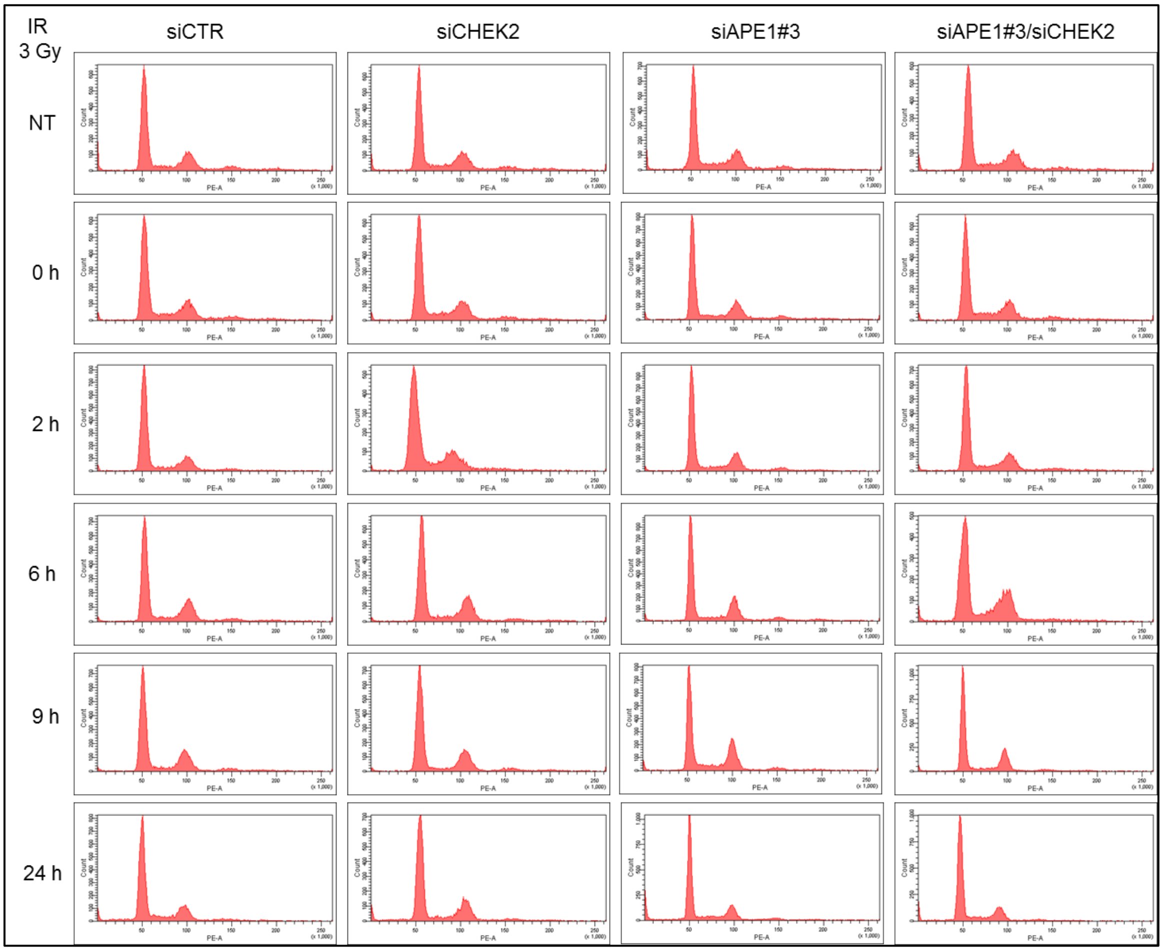

B

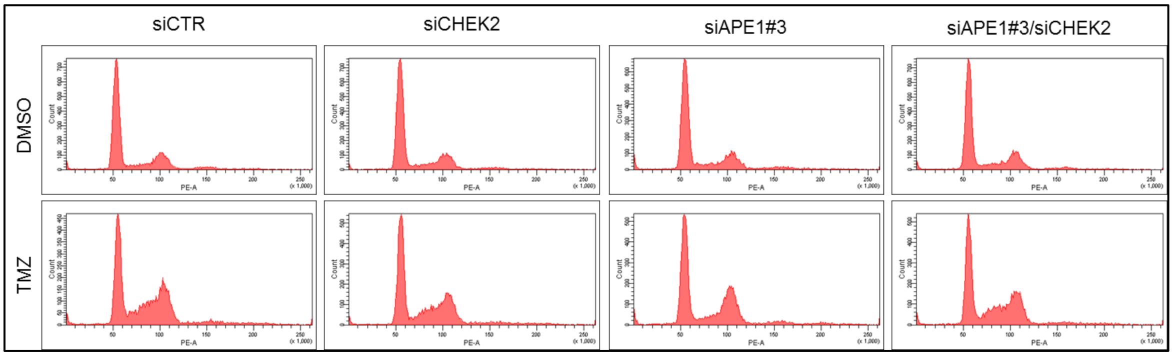

Figure S7. Cell cycle distribution of Ape1 and/or Chk2-depleted U251-MG cells after DNA damage (A) U251-MG cells were transfected with the indicated siRNAs. 48 h after the transfection, cells were exposed to ionizing radiation (IR, 3 Gy), harvested at the indicated time points, and subjected to cell cycle analysis. **(B)** U251-MG cells were treated with DMSO (control) or temozolomide (TMZ, 1000 μM) for 1 h at 48 h of siRNA transfection. Cells were washed and incubated for another 16 h prior to the fixation for cell cycle analysis.

Figure S8, related to Figures 3-6

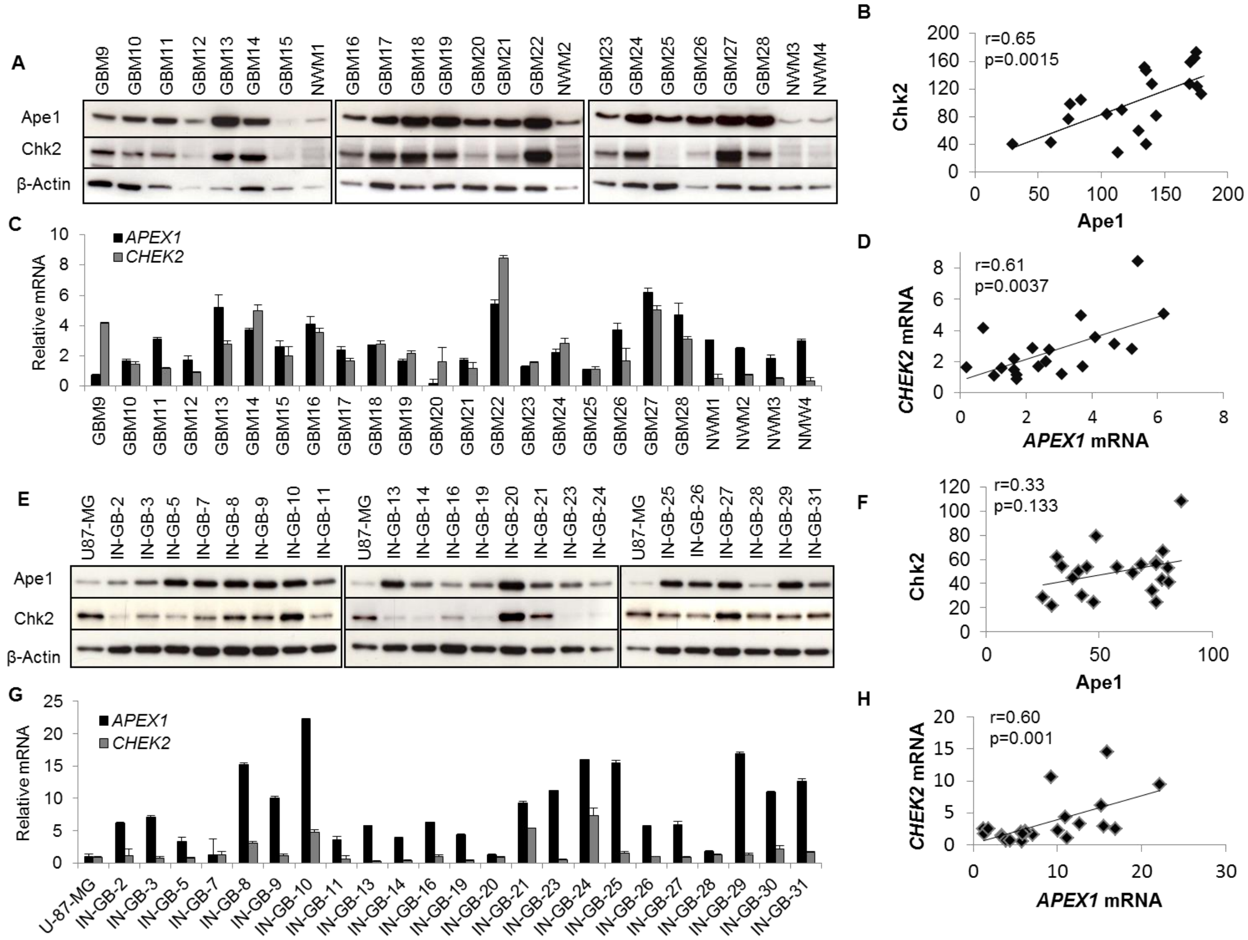

Figure S8. Coordinated expression of Ape1 and Chk2 in glioblastoma tumor tissues and in patient-derived early passage glioblastoma cells. (A) Western blot analyses of glioblastoma tissue lysates were performed with antibodies to Ape1, Chk2, and β-actin. **(B)** Quantified relative (to β-actin) band intensities were subjected to the Pearson Correlation Analysis, and correlation coefficient (r) was calculated, r=0.65; p=0.0015; n=20. NWM; Normal White Matter. **(C)** Gene expression levels of *APEX1* and *CHEK2* in glioblastoma tumor tissues. Total RNA was isolated from tissue samples, and real-time PCR was performed. Expression levels of *APEX1* and *CHEK2* mRNAs were normalized to GAPDH. APEX1 level for NMW1 was set as 1.0. (**D**) Pearson correlation coefficient (r) calculated for *APEX1* and *CHEK2* mRNA is shown, r=0.61; p=0.0037; n=20. **(E)** Immunoblots were performed in the early passage glioblastoma cells for Ape1, Chk2, and β-Actin. A commonly used GBM cell line, U87-MG was also included in the study. (**F**) Pearson correlation coefficients (r) for protein were calculated (r=0.33; p=0.133; n=22). **(G)** Transcription levels of *APEX1* and *CHEK2* in early passage glioblastoma cells. Total RNAs were isolated from the cells, and real-time PCR was performed for *APEX1*, *CHEK2*, and *GAPDH*. The mRNA expression levels in each sample were normalized to the levels of *GAPDH*. (**H**) Pearson correlation coefficients (r) for mRNA (r=0.60; p=0.001; n=23) levels of *APEX1* and *CHEK2* in glioblastoma cell cultures are shown.

Figure S9, related to Figures 3-6

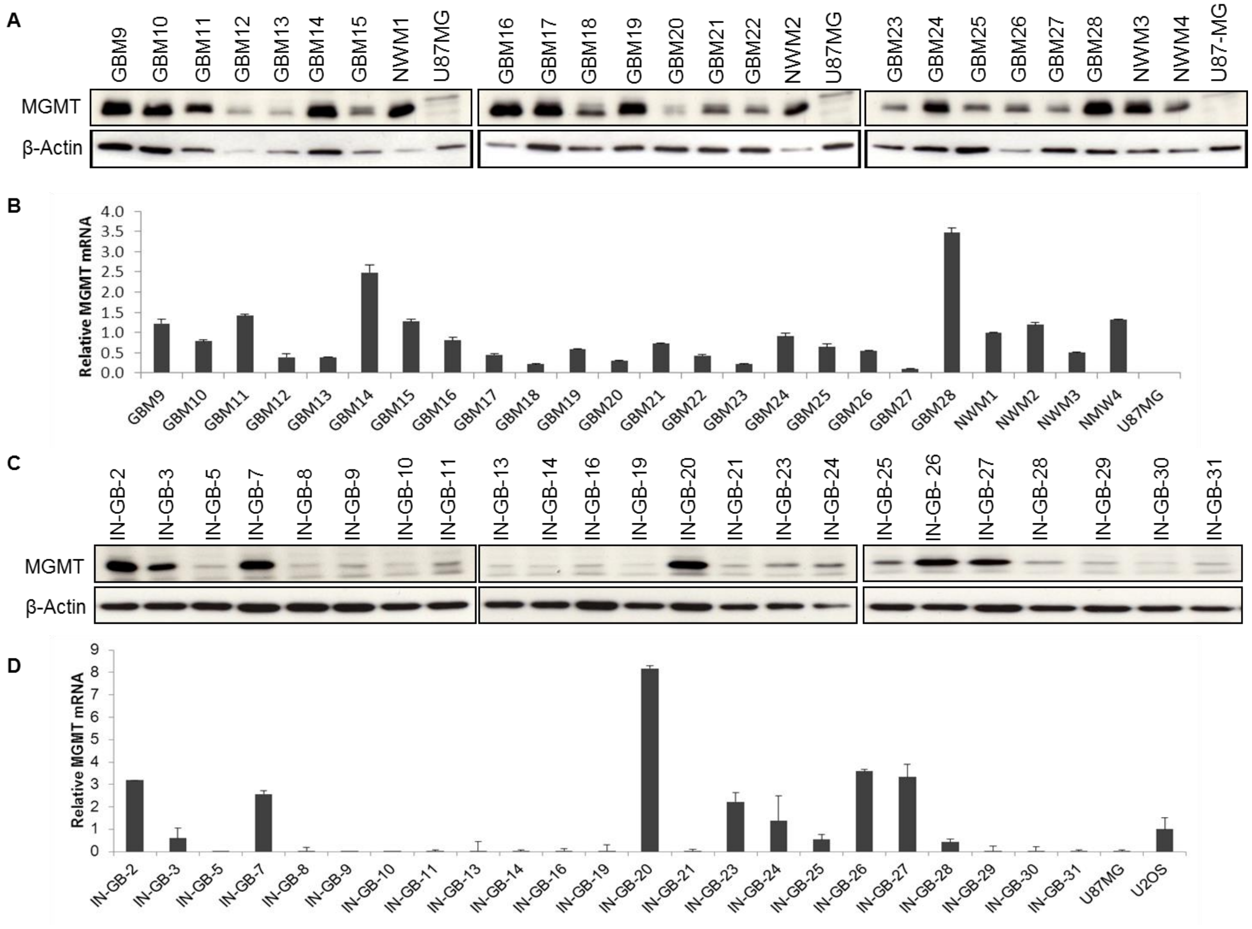

Figure S9. MGMT expression levels in glioblastoma tissues and cell cultures. (A) MGMT protein levels in glioblastoma tissues**.** Immunoblots were performed with anti-MGMT and anti-β-actin in glioblastoma tissue lysates. (**B**) *MGMT* gene expression in glioblastoma tissues. Total RNAs were isolated from glioblastoma tissue samples, and analyzed by real-time PCR for *MGMT* mRNA expression. The data were normalized to *GAPDH* expression in each sample (±SD). (**C)** MGMT protein levels in patient-derived early passage glioblastoma cells. MGMT protein levels were detected by immunoblotting in glioblastoma cell cultures. β-Actin was used as loading control. **(D)** *MGMT* gene expression in patient-derived early passage glioblastoma cells. Total RNAs were isolated from patient-derived early passage glioblastoma cells, and analyzed by real-time PCR for *MGMT* gene expression. The normalized values to *GAPDH* are shown (±SD).

Figure S10, related to Figures 4-6

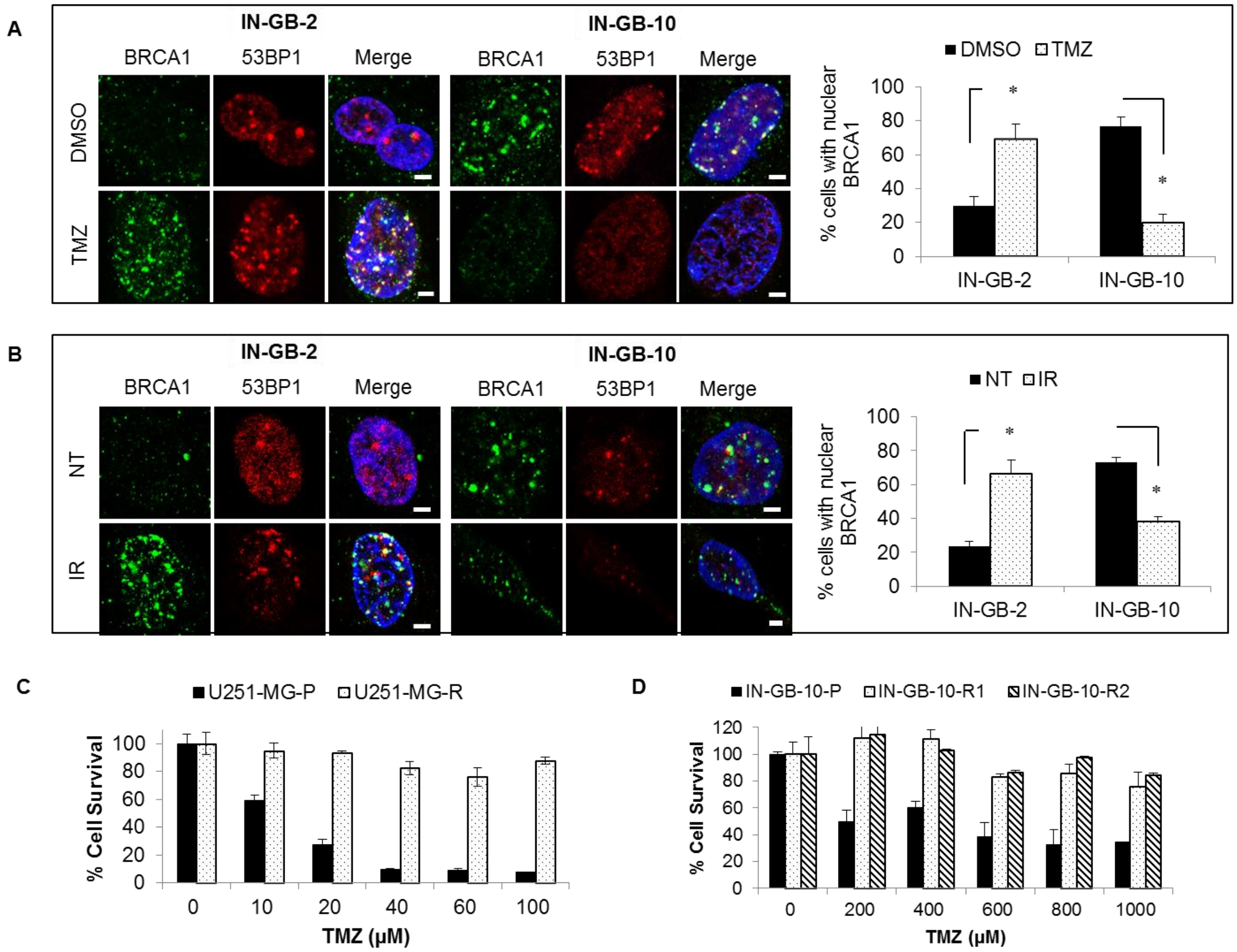

Figure S10. Intrinsically TMZ-resistant early-passage patient cells show increased HR activity upon exposure to DNA damage. (A) IN-GB-2 and IN-GB-10 cells were treated with DMSO or TMZ (1000 µM) for 1h. 24 h later, cells were fixed and immunostained with BRCA1 and 53BP1 antibodies. At least 300 cells from three independent experiments were counted, and the cells showing nuclear BRCA1 staining (including foci formation or diffuse nuclear staining) were quantified. *p<0.01; Student's *t* test. Scale bar, 5 µM. (**B**) IN-GB-2 and IN-GB-10 cells were exposed to IR (3 Gy), and cultured for another 9 h. Cells were then fixed and immunostained with BRCA1 and 53BP1 antibodies. At least 300 cells from three independent experiments were counted, and the cells showing nuclear BRCA1 staining (including foci formation or diffuse nuclear staining) were quantified. *p<0.01; Student's *t* test. Scale bar, 5 µm. **(C)** Generation of TMZ-resistant glioblastoma cells. U251-MG cells were exposed to increasing concentrations of TMZ (10-320 µM) in a period of three months. One clone (U251-MG-R) propagating in the presence of 320 µM of TMZ was isolated and cultured with TMZ for at least a month prior to the study. The parental (U251-MG-P) and resistant (U251-MG-R) cells were exposed to the indicated concentrations of TMZ. Cells were counted three days after the exposure, and percentages of cell survivals were calculated. **(D)** Intrinsically TMZ-sensitive IN-GB-10 cells were exposed to increasing concentrations of TMZ (10-320 µM) for three months. Two clones, IN-GB-10-R1 and IN-GB-10-R2, were isolated, and percentages of cell survivals were calculated after TMZ exposure as described in C.

Figure S11, related to Figures 4-6

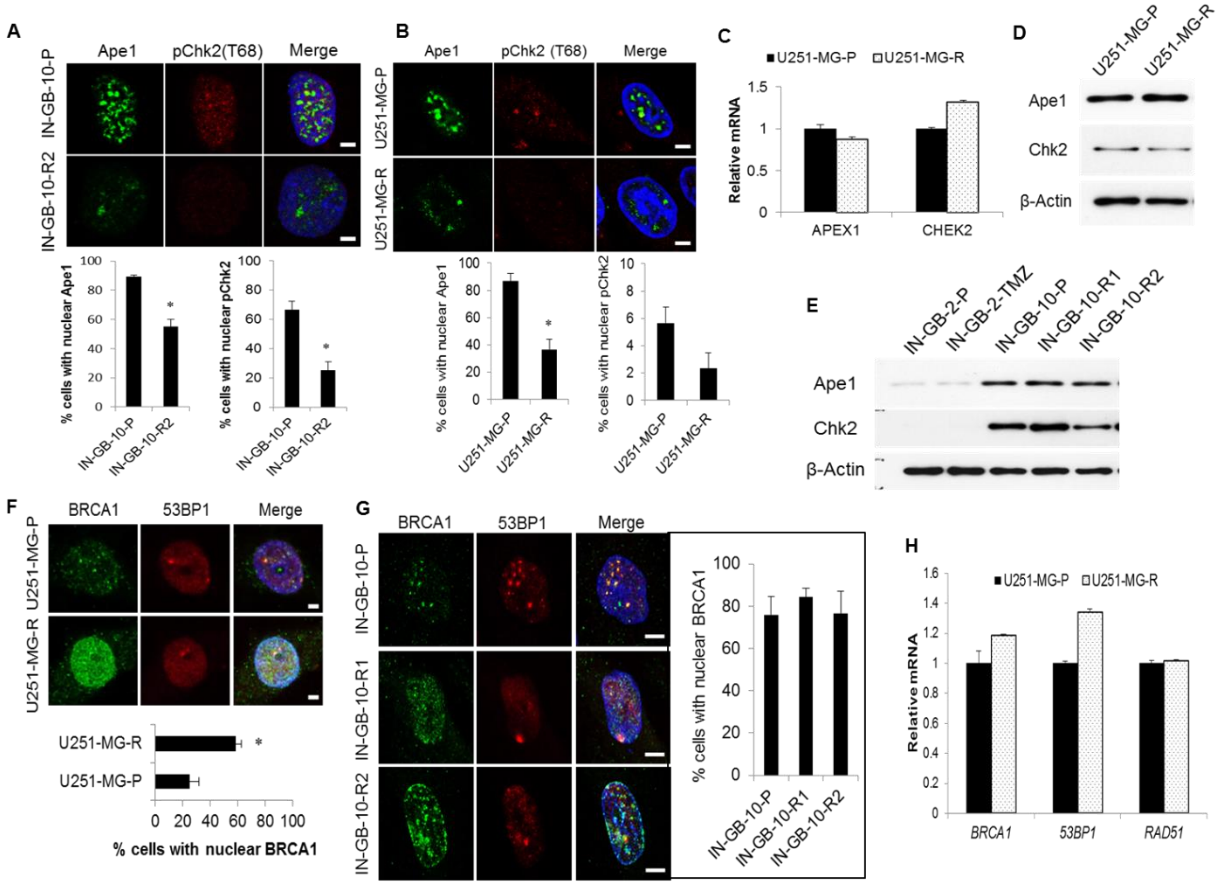

Figure S11. Nuclear localizations of Ape1, pChk2 (Thr 68), and BRCA1 are altered in TMZ-resistant U251-MG cells. (A) IN-GB-10 parental (IN-GB-10-P) and its resistant clone IN-GB-10-R2 cells were fixed and immunostained with Ape1 and pChk2 (Thr 68) antibodies. At least 300 cells from three independent experiments were counted, and the cells showing nuclear staining (including foci formation or diffuse nuclear staining) were quantified. *p<0.01; Student's *t* test. Scale bar, 5 µm. **(B)** U251-MG parental (U251-MG-P) and its resistant cell clone (U251-MG-R) were immuno-stained for Ape1 and pChk2 (Thr 68). At least 300 cells from three independent experiments were counted and percentages of cells showing nuclear Ape1 or pChk2 (Thr 68) staining (including foci formation and marked diffuse nuclear staining) were calculated. *p<0.01; Student's *t* test. Scale bar, 5 µm. (**C)** Transcriptional gene regulation of *APEX1* and *CHEK2* in parental and TMZ-resistant U251-MG cells. **(D)** Protein expression levels of Ape1 and Chk2 are comparable in U251-MG-parental and U251-MG-resistant cells, as well as **(E)** in IN-GB-10-parental and its two different resistant clones. IN-GB-2-parental and IN-GB-2-TMZ-treated cells express much lower levels of Ape1 and Chk2. **(F)** U251-MG-P and U251-MG-R cells were immuno-stained for BRCA1 and 53BP1. Scale bar, 5 µm. At least 300 cells from three independent experiments were counted and percentage of cells showing nuclear BRCA1 staining (comprising foci formation and marked diffuse nuclear staining) was calculated. *p<0.01; Student's *t* test. (**G)** Parental IN-GB-10-P and its TMZ-resistant cell clones IN-GB-10-R1 and IN-GB-10-R2 were immunostained for BRCA1 and 53BP1. At least 300 cells from three independent experiments were counted and percentage of cells showing nuclear BRCA1 staining (including foci formation and marked diffuse nuclear staining) was calculated. Scale bar, 5 µm. **(H)** Transcriptional gene regulation of *BRCA1*, *53BP1* and *RAD51* in parental and TMZ-resistant U251-MG cells.

SUPPLEMENTAL EXPERIMENTAL PROCEDURES

Cell culture and chemicals

Human osteosarcoma cells U2OS (ATCC), HEK293 (ATCC), U251-MG, U87-MG (ATCC), and patient-derived early passage glioblastoma cells were grown in Dulbecco's Modified Eagle's Medium (DMEM), containing 10% fetal bovine serum. Cells were treated with DMSO (Sigma-Aldrich), BSA (Roche), temozolomide (TMZ) (Sigma-Aldrich), methyl methane sulfonate (MMS) (Sigma-Aldrich), etoposide (Sigma-Aldrich), hydroxyurea (Sigma-Aldrich), thymidine (Sigma Aldrich), as indicated.

Cell Viability Assay

U251-MG cells were transfected with the siRNAs. Next day, cells were replaced with fresh media and incubated for another 8 h. Cells were then seeded onto 6-well plates at the density of 150,000 cells/well. Cells were counted after staining with trypan blue (0.4%, Invitrogen) by using a Neubauer cell counting chamber. Percentage of cell viability was calculated relative to the control-siRNA treated cells. Error bars indicate standard deviations obtained from three independent experiments. $p<0.01$; Student's *t* test.

Cycloheximide Chase Assay

U251-MG cells were transfected with siRNAs. At 48 h of the transfection, cells were treated with cycloheximide (CHX, 50 µg/ml) for the indicated time points. Cells were then harvested and subjected to Western blot for Ape1 and Chk2 protein levels. β-Actin was used as loading control. In order to investigate the effect of DNA damage on the half-lives of Ape1 and Chk2, U251-MG cells were treated with DMSO (control) or temozolomide (TMZ, 1000 µM) for 1 h. After several washing cycles, cells were treated with CHX (50 µg/ml). Cells were then harvested at the indicated time points, and subjected to Western blot.

Microirradiation

U251-MG cells were seeded into 6-well plates at a density of $5x10^5$ cells. Cells were infected with the lentiviral particles carrying GFP-tagged Ape1 wild-type and domain-mutant sequences of Ape1, as described earlier [1]. After selection with G418 (400µg/ml) for at least one week, cells were seeded into IBIDI µ-slide wells (10^3cells/well), and transfected with a RFP-tagged PCNA plasmid [2] by using Lipofectamine-2000 transfection reagent (Invitrogen) according to the manufacturer's instructions. 48 h post-transfection, cells were exposed to laser microirradiation by using an inverse spinning disc confocal microscope unit, equipped with CW lasers (405, 488, 561 or 640 nm laser) and a pulsed 355 nm laser diode (BioOptics Core Facility of the MFPL, University of Vienna). Cells were pulsed with 20% intensity of 355nm laser, and live-imaging of fluorescence signals at incision sites was performed by using 488 nm laser (GFP) and 561 nm laser (RFP) for 30 min. At least three independent laser incisions were performed for each cell type expressing wild-type or mutant variants of Ape1. Images were analyzed by the VisiView software. For immunofluorescence experiments, U2OS cells were exposed to laser microirradiation at 20% intensity of 355 nm laser, washed with PBS immediately, and fixed with methanol within 2 min of laser incisions. Cells were then immunostained for endogenous Ape1 and γH2AX. DNA was labelled with DAPI. At least three independent laser incisions were performed. Images were taken by confocal laser scanning microscopy (LSM780, Zeiss), and analyzed by ZEN software (Zeiss). Absolute fluorescence intensities of the laser-microirradiated areas were calculated by subtracting the cellular background fluorescence from the laser-induced damage site fluorescence for each time-point (Image J, NIH). Error bars represent standard deviation of three independent measurements.

BrdU incorporation assay

48 h after siRNA transfection, cells were pulse-labeled with 10 µM BrdU for 40 min at 37˚C. Incorporation of BrdU was measured with the FITC BrdU Flow Kit (BD Pharmingen). Data were analyzed using BD FACS Diva Software v6.1.2.

Immunohistochemistry

Immunohistochemistry was performed on formalin-fixed, paraffin-embedded (FFPE) tumor tissues cut at a thickness of 3 µm using anti-Rad51 antibody (Rad51 polyclonal rabbit IgG, 1:100, antigen retrieval, pH9, Sigma Life Science, St. Louis, US) and EnVisionTM FLEX+ (Dako, Glostrup, Denmark) as visualization system. Human testicle served as positive control. Whole slides were evaluated for nuclear tumor cell immunoreactivity, expressed as percentage. Changes in nuclear Rad51 expression in matched primary and recurrent tumor tissues were assessed using the Wilcoxon signed-rank sum test ($p < 0.001$).

Western Blot, Co-immunoprecipitation Studies, and Chromatin isolation

Whole cell and tissue lysates were prepared in lysis buffer (10 mM Tris-HCl pH 7.4, 150 mM NaCl, 5 mM EDTA, pH 8.0, 1% Triton X-100) containing protease inhibitors (Roche). Protein concentrations were determined by Bradford assay (Bio-Rad), and samples containing equivalent amounts of protein (20-30 µg) were subjected to SDS-PAGE. Immunoblotting was carried out with the antibodies detecting Ape1 (Santa Cruz, sc-17774), Chk2 (Cell Signaling, #3440 or Santa Cruz, sc-56296), phospho-Chk2 (Thr68) (Cell Signaling, #2661), phospho-Chk1 (Ser345) (Cell Signaling, #2348), Histone H3 (Cell Signaling, #9715), MGMT (Millipore, #MAB16200), Rad51 (Sigma, HPA039310), BRCA1 (Millipore, OP92), and β-actin (Santa Cruz, sc-47778). The blots were either developed with ECL reagent (GE-Amersham), or scanned using a Licor Odyssey Scanner (Fig. 1C). Image quantifications were performed using Totallab software. For co-immunoprecipitation studies, HEK293 cells were treated with BSA (0.5 %; 24 h), double thymidine (DT, 2 mM), hydroxyurea (HU, 2 mM; 16 h), or methyl methane sulphonate (MMS, 250 µM; 4 h), along with asynchronized (Asyn.) cells. MMS-treated cells were washed with PBS several times, and incubated for another 12 h before harvesting. In another set of co-immunoprecipitation reactions, HEK293 cells were treated with temozolomide (TMZ, 250 µg/ml; 4 h) or exposed to ionizing radiation (IR, 3 Gy). TMZ-treated cells were washed with PBS several times, and incubated at 37 °C for another 4 h prior to the harvest. Following the IR exposure, cells were immediately returned to the incubator and harvested 6 h after the IR exposure. Cells were then subjected to immunoprecipitation (IP) with a Chk2 antibody. HEK293 cell extracts were prepared in IP lysis buffer (50 mM Tris-HCl pH 7.5, 150 mM NaCl, 1 mM EDTA, 1% NP-40) supplemented with protease inhibitors, and benzonase (25 U/reaction, Novagen) to digest DNA. Cell lysates (500 µg protein/reaction) were incubated with the antibodies (3 µg/reaction) for Chk2 (Santa Cruz, sc-56296) for 4 h at 4 °C. A mouse IgG was used as control, and immune complexes were collected by using Protein A/G-coupled agarose beads after o/n incubations at 4°C. Immunoprecipitates were washed 4 times in IP lysis buffer, and boiled in 5xSDS loading buffer (0.25 M Tris-HCl, pH 6.8, 15% SDS, 50% glycerol, 25% β-mercaptoethanol, 0.01% bromophenol blue). After the incubation with antibodies for Ape1 or Chk2, blots were developed with ECL reagent (GE-Amersham). Chromatin isolation was performed as described earlier [3]. Chromatin fractions were boiled in SDS buffer, and analyzed by Western blot.

Preparation of Metaphase Chromosomes

RNAi-treated U251-MG cells were treated with DMSO (control) or TMZ (1000 µM, 1 h) at 48 h of siRNA transfection. After extensive washing, cells were treated with nocodazole (Sigma) (100 ng/ml) for 16 h, and mitotic cells were harvested by mitotic shake-off. Metaphase chromosomes were prepared according to the standard protocol. Briefly, cells were incubated with 0.56 M KCL at room temperature, followed by several cycles of fixation by Carnoy fixative (3:1 methanol:glacial acetic acid), and dropped onto slides. Chromosomes were stained with DAPI, and analyzed for chromosome fusions by confocal laser scanning microscopy (LSM780, Zeiss). Metaphase spreads from three independent experiments were analyzed, and chromosome fusions per metaphase were counted. Standard deviations were calculated by counting at least 50 metaphases for each treatment. Significance between the groups was analyzed by Student's *t* test.

Frequency of Sister Chromatid Exchange (SCE)

Frequency of SCE was determined in Ape1 and/or Chk2-downregulated U2OS cells as described previously [4], with small modifications. Briefly, 16 h after siRNA treatments, cells were synchronized with a single round of thymidine treatment for 24 h. After the release from thymidine, 10 µM BrdU was added into the culture at 48 h of the

transfection, and incubated for another 24 h. Next day, cells were treated with 350 µM MMS for 3 h. After extensive washing steps, cells were replaced with fresh media containing 10 µM BrdU and 100 ng/ml nocodazole, and incubated for another 16 h. Metaphase chromosomes were stained with Giemsa. Representative images are shown. The number of chromosomes showing SCE per metaphase cell (SCE per cell) was quantified by analysis of at least thirty metaphase cells per sample. Medians of the number of chromosomes with SCE were presented in a box plot chart. ***p<0.0001; (siAPE1#3 vs. siAPE1#3/siCHEK2); Student's *t* test.

Orthotopic Glioblastoma Mouse Model

Animal experiments were approved by the Committee on Animal Experiments of the VU University Amsterdam, the Netherlands. The U251 orthotopic glioblastoma mouse models were generated via injection of U251-FM glioblastoma cells stably expressing Firefly luciferase (Fluc) and mCherry [5]. Prior to injection, U251-FM cells were transfected with the following siRNAs: CTR-siRNA, APE1-siRNA#3, CHEK2-siRNA, and combination of APE1-siRNA#3 and CHEK2-siRNA using Lipofectamine-2000 reagent (Invitrogen), according to the manufacturer's instructions. 3 hours after the transfection, medium was replaced, and cells were cultured for 16 hours. Cells were then trypsinized, and concentrated to a density of 10^8 cells/ml before the orthotopical injection into the striatum of female athymic Nude-Foxn1nu mice (age 8-9 weeks; Harlan, Horst, The Netherlands). For stereotactic surgery, mice were sedated using gas anesthesia (1.5 L O2/minute and 2.5% isoflurane) and mounted into a stereotactic frame as described earlier [6]. A 0.8 mm burr hole was opened at 2 mm lateral, 0.5 mm posterior from the bregma. Cells were slowly injected with a Hamilton syringe at a depth of 3 mm. The skin was closed with surgical glue and the animals returned to their cages. Mice were sacrificed upon discomfort or loss of more than 15% body weight. Tumor growth was monitored by bioluminescence imaging (BLI); Luciferin (5 mg) was administered in mice intraperitoneally, and bioluminescence signal was measured after 10 minutes by a charge-coupled device (CCD camera) (IVIS lumina system) under the condition of isoflurane inhalation anesthesia. Mice were treated with temozolomide (10 mg/kg) at day 2, day 3 and day 4 after intracranial injection of the tumor cells. Temozolomide was dissolved in saline (1 mg/ml) and administered in mice intraperitoneally. For data presentation, group means of BLI were calculated. In the cases where animal died, the last value was forwarded for better visualization of the data. Statistics were performed by two-sided t-test analysis for equality of means on each monitoring day (IBM SPSS statistics vs. 20). Missing data were omitted for this statistical analysis. Kaplan-Meier survival curves were plotted by using GraphPad Prism 5.

HR Assay

U2OS-DR cells containing a single copy of the DR-GFP reporter were transfected with the indicated siRNAs, along with the I-Sce I endonuclease expression vector pCBASce [7]. Cells were harvested 48 h after transfection, and GFP-positive cells were scored by a flow cytometry (BD Biosciences-LSRFortessa), and analyzed by the FACSDiva software. Patient-derived early passage glioblastoma cells, IN-GB-2 and IN-GB-10 were electroporated with the DR-GFP reporter and the pCBASce vector. A GFP expression vector (pIRES-neo-EGFP, Clontech) was employed to assess transfection efficiency: GFP-positive cells were scored as ~80 % for IN-GB-2 cells, and ~40% for IN-GB-10 cells after three days following transfection with the GFP expression vector. Cells were treated with DMSO (control) or TMZ (1000 µM) for 1 h. After extensive wash, cells were cultured for another 24 h, and GFP-positive cells were quantified by a flow cytometry. Each experiment was performed at least three times, and significance of the results was analyzed by Student's *t* test.

NHEJ Assay

H1299dA3-1 cells stably carrying the IRES-TK-EGFP DNA reporter were transfected with the indicated siRNAs, along with an I-SceI expression vector. GFP-positive cells were counted at 72 h post-transfection by flow cytometry as described earlier [8].

RNA isolation and quantitative real-time PCR

RNA samples were prepared from tumor tissues or normal-appearing white matter using Trizol. For real-time PCR, 1 µg of total RNA was reverse transcribed using the SuperScript First-Strand Synthesis System (Invitrogen; 11904018). Quantitative real-time PCR was performed using the Applied Biosystem SYBR Green master mix in an iCycler RT-PCR System (Applied Biosystems). The data were normalized for GAPDH as internal control. The following primer sequences (Integrated DNA Technologies, IDT) were used in the study: APEX1, forward 5'-

CAATACTGGTCAGCTCCTTCG-3’ and reverse 5’-CAAATTCAGCCACAATCACCC-3’; CHEK2, forward 5’-GCGCCTGAAGTTCTTGTTTC-3’ and reverse 5’-GTCCTATGCTCAGAGAAAGGTG-3’; BRCA1, forward 5’-GCCTTCTAACAGCTACCCTTC-3’ and reverse 5’– CTTCTGGATTCTGGCTTATAG-3’; 53BP1, forward 5’-GGAACAGAAGGAGAAAGAGAAGG-3’ and reverse 5’-CAGAACCCCAAAACCCAAC-3’; RAD51, forward 5’-GTGGTAGCTCAAGTGGATGG-3’ and reverse 5’-GGGAGAGTCGTAGATTTTGCAG-3’; MGMT, forward 5’-GCTGAATGCCTATTTCCACC-3’ and reverse 5’- CACTTCTCCGAATTTCACAACC -3’; GAPDH, forward 5’- ACATCGCTCAGACACCATG-3’ and reverse 5’-TGTAGTTGAGGTCAATGAAGGG-3’.

TCGA Data Analysis

The TCGA mRNA expression microarray data (AgilentG4502A_07 log2 tumor/normal ratio) for glioblastoma patients were downloaded from the TCGA Data Portal (http://tcga-data.nci.nih.gov/tcga). The correlation between *APEX1* and *CHEK2* in the Tumor Glioblastoma-TCGA-540-MAS5.0-u133a dataset was analyzed by using the R2: Microarray Analysis and Visualization Platform (http://r2.amc.nl). Survival analyses were performed using the R software platform (http://www.R-project.org).

Supplementary References

1. Madlener, S., *et al.* Essential role for mammalian apurinic/apyrimidinic (AP) endonuclease Ape1/Ref-1 in telomere maintenance. *Proc Natl Acad Sci U S A* **110**, 17844-17849 (2013).
2. Sporbert, A., Domaing, P., Leonhardt, H. & Cardoso, M.C. PCNA acts as a stationary loading platform for transiently interacting Okazaki fragment maturation proteins. *Nucleic Acids Res* **33**, 3521-3528 (2005).
3. Wysocka, J., Reilly, P.T. & Herr, W. Loss of HCF-1-chromatin association precedes temperature-induced growth arrest of tsBN67 cells. *Mol Cell Biol* **21**, 3820-3829 (2001).
4. Goto, K., Maeda, S., Kano, Y. & Sugiyama, T. Factors involved in differential Giemsa-staining of sister chromatids. *Chromosoma* **66**, 351-359 (1978).
5. Mir, S.E., *et al.* In silico analysis of kinase expression identifies WEE1 as a gatekeeper against mitotic catastrophe in glioblastoma. *Cancer Cell* **18**, 244-257 (2010).
6. Caretti, V., *et al.* Monitoring of tumor growth and post-irradiation recurrence in a diffuse intrinsic pontine glioma mouse model. *Brain Pathol* **21**, 441-451 (2011).
7. Pierce, A.J., Johnson, R.D., Thompson, L.H. & Jasin, M. XRCC3 promotes homology-directed repair of DNA damage in mammalian cells. *Genes Dev* **13**, 2633-2638 (1999).
8. Ogiwara, H., *et al.* Histone acetylation by CBP and p300 at double-strand break sites facilitates SWI/SNF chromatin remodeling and the recruitment of non-homologous end joining factors. *Oncogene* **30**, 2135-2146 (2011).
